# Role of FRG1 in predicting the overall survivability in cancers using multivariate based optimal model

**DOI:** 10.1038/s41598-021-01665-w

**Published:** 2021-11-18

**Authors:** Rehan Khan, Ananya Palo, Manjusha Dixit

**Affiliations:** 1 School of Biological Sciences, National Institute of Science Education and Research, Bhubaneswar, HBNI, P.O. Jatni, Khurda, 752050 Odisha India; 2 School of Biological Sciences, NISER, Room No.- 203, P.O. Jatni, Khurda, Odisha 752050 India

**Keywords:** Cancer, Breast cancer, Cancer epidemiology, Cancer models, Gastrointestinal cancer, Lung cancer

## Abstract

FRG1 has a role in tumorigenesis and angiogenesis. Our preliminary analysis showed that FRG1 mRNA expression is associated with overall survival (OS) in certain cancers, but the effect varies. In cervix and gastric cancers, we found a clear difference in the OS between the low and high FRG1 mRNA expression groups, but the difference was not prominent in breast, lung, and liver cancers. We hypothesized that FRG1 expression level could affect the functionality of the correlated genes or vice versa, which might mask the effect of a single gene on the OS analysis in cancer patients. We used the multivariate Cox regression, risk score, and Kaplan Meier analyses to determine OS in a multigene model. STRING, Cytoscape, HIPPIE, Gene Ontology, and DAVID (KEGG) were used to deduce FRG1 associated pathways. In breast, lung, and liver cancers, we found a distinct difference in the OS between the low and high FRG1 mRNA expression groups in the multigene model, suggesting an independent role of FRG1 in survival. Risk scores were calculated based upon regression coefficients in the multigene model. Low and high-risk score groups showed a significant difference in the FRG1 mRNA expression level and OS. HPF1, RPL34, and EXOSC9 were the most common genes present in FRG1 associated pathways across the cancer types. Validation of the effect of FRG1 mRNA expression level on these genes by qRT-PCR supports that FRG1 might be an upstream regulator of their expression. These genes may have multiple regulators, which also affect their expression, leading to the masking effect in the survival analysis. In conclusion, our study highlights the role of FRG1 in the survivability of cancer patients in tissue-specific manner and the use of multigene models in prognosis.

## Introduction

FSHD Region Gene 1 (FRG1) gene is present on human chromosome 4q35. Being the primary candidate gene of Facioscapulohumeral Muscular Dystrophy (FSHD), a disease related to muscle weakness and atrophy, studies pertaining to FRG1 primarily focused on muscles^1^. While the exact function of FRG1 is yet to be deciphered, various studies have indicated its role in mRNA splicing^2^. The biochemical activity analysis of human FRG1 revealed RNA binding and actin-binding properties which have direct implications in RNA biogenesis, transport, and cytoplasmic localization^3^. The recent 3D cryo-EM structure of the human spliceosomal C complex has shown that the FRG1 is a part of spliceosome machinery, which can have multiple prospects on gene expression regulation^4^. The first clue regarding the role of FRG1 in angiogenesis or tumorigenesis came from a study in *X. laevis* where an increase in branching and vasculature was observed by overexpressing FRG1^5^. Our research group, for the first time, showed reduced expression of FRG1 in cancer tissues. FRG1 affected the proliferation, migration, invasion, and angiogenic potential of cancer cell lines and the expression of G-CSF and MMP10^6^. Reduced FRG1 expression in androgen receptor negative prostate cancer cell lines increased invasiveness and migratory properties^7^.

Although our previous study showed that FRG1 affects EMT, yet its role in survival is not clear. Our preliminary analysis did not indicate the robust effect of FRG1 on overall cancer survival in all cancer types. FRG1 in conjunction with other genes may affect the survival of cancer patients. Alternatively, other genes which are also altered in cancers conceal the analysis of the effect of FRG1 on the OS.

We first determined the genes positively correlated with FRG1 using multiple databases in different cancer types, based on this hypothesis. We performed Cox regression analysis to come up with the model which predicts cancer survival significantly. Later we used these genes to determine the pathways in which the FRG1 is involved. Common genes which were part of the FRG1 related pathways in different cancers were experimentally validated. Our study shows the importance of the use of multigene models in survival prediction.

## Material and methods

### Workflow

**Figure 1:**
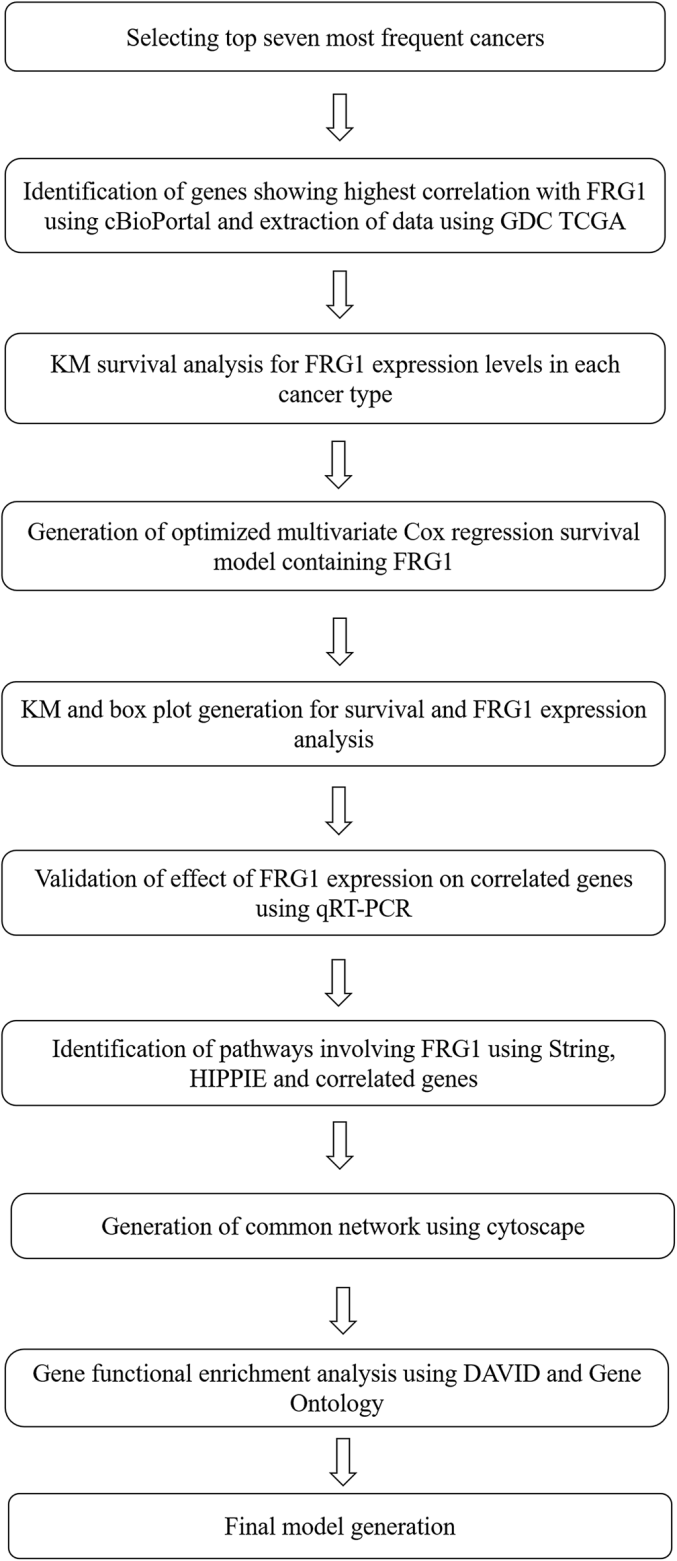
Flow chart of the study design.

We chose the top seven cancers with the highest incidence For this study based on Global Cancer Observatory data^8^. FRG1 co-expression data of the top 20 most correlated genes was obtained from cBioPortal^9,10^. mRNA expression and clinical datasets for all the patients in each cancer type were downloaded from Genomic Data Commons (GDC) Data Portal (Htseq-FPKM-UQ)^11^. Figure 1 shows the workflow for the complete analysis. Kaplan Meier survival analysis was performed in each cancer type to observe the effect of FRG1 mRNA expression on the survivability of patients^12^. Stratified multivariate cox regression^13^ was performed to determine the association between overall patient survival and gene expression levels of FRG1 along with the 20 correlated genes (top correlated genes based on the spearman’s correlation, r_s_) in all the cancer types. The model was optimized by removing the least correlated genes sequentially till the FRG1 was significant. The risk score was calculated for each patient, and the patients were divided into low and high-risk groups based on the median risk score^14,15^. We created Kaplan–Meier plots to identify the difference in survival between the low and high-risk groups in different cancer types. Box plots were created to represent the FRG1 expression in both the risk groups.

Using STRING^16^ and HIPPIE^17^ web tools, we developed a network of FRG1 and the top correlated genes to find the known interactions at various levels. From the cancer type-specific pathways, a common pathway was identified. The effect of FRG1 expression on these common genes was validated via RT-PCR. Using DAVID (KEGG pathway)^18,19^and Gene Ontology (GO)^20^ we performed gene functional enrichment analysis to identify potential biological processes, molecular function and signaling pathways involved in different cancer types.

### Data sources and processing

Co-expression data of FRG1 was obtained from cBioPortal (accessed on 20 Aug 2020). In cBioPortal for each cancer type “TCGA, Firehose Legacy” database was selected for correlation analysis. In co-expression tab FRG1 correlated genes were searched using data for mRNA expression (RNA Seq V2 RSEM).

For survival analysis, data of expression profiles along with clinical data was downloaded from GDC Data Portal (up to 19 Dec 2020) for all the cancer types. Settings chosen to download the data from GDC TCGA were as follows; Data Category- Transcriptome Profiling, Data type- Gene Expression Quantification, Experimental Strategy- RNA-Seq, Primary site- Cancer Type, Program- TCGA, Workflow Types- Htseq-FPKM-UQ.

### Overall survival analysis for single gene

To test the effect of FRG1 expression on the survivability of patients in our chosen cancer types, we performed the Kaplan Meir survival analysis using the TCGA data downloaded from GDC. We did the analysis in R using the "survival" and "survminer" libraries. To determine the optimal cut-off point of FRG1 expression for KM plot, we used the surv_cutpoint() function and plotted using plot() (Supplementary Fig. S1). KM plot were made using ggsurvplot() function.

The Kaplan–Meier plotter (KM-Plotter)^21^ was used to validate the effect of FRG1 mRNA expression on OS. In the KM plotter, under “Start KM Plotter for pan-cancer” tab, dataset for specific cancer was selected. At “Gene symbol” FRG1 was given as input. Patients were split according to the cutoffs used in the TCGA data analysis.

### Survival analysis and identification of prognostic genes

We analyzed the correlation between OS time and gene expression by using stratified multivariate Cox regression. The model was optimized by removing the least correlated genes until the FRG1 remained significant. A risk score was calculated for each patient based on the following equation,

$$Risk \; score={\sum }_{i=1}^{n}{exp}_{i}{\beta }_{i}$$

where n was the number of prognostic genes, exp_i_ the expression value of gene i, and β_i_ the regression coefficient of gene i in the Cox regression analysis. Patients were classified into high- and low-risk groups, using the median risk score as a cutoff value. Box plots were generated to compare the FRG1 mRNA expression level between the low and high-risk groups. The Log-Rank test was used to determine the statistical significance of the difference in OS between the two groups.

### Pathway analysis

Search Tool for the Retrieval of Interacting Genes/Proteins (STRING) is a biological database and web resource of known and predicted protein–protein interactions (PPI). For each cancer type, a model was created using STRING PPI network data. In the model, each node is represented by a protein and, edges show physical interaction between the two proteins. The missing links between FRG1 and co-expressed genes were found using Human Integrated Protein–Protein Interaction rEference (HIPPIE), which is based on the earlier reports of FRG1 interacting proteins. Cytoscape (Version: 3.8.2) was used to visualize the networks and to find the intersection of all the pathways using the merge tool^22^. We did GO Enrichment analysis for the identification of enriched biological processes and molecular functions^20^. Database for Annotation, Visualization, and Integrated Discovery (DAVID)^19^ was used to find out the KEGG (Kyoto Encyclopedia of Genes and Genomes) pathways for the set of FRG1 correlated genes in different cancer types^18^.

### Cell line and Western blot

Human embryonic kidney (HEK293T) cells were obtained from the cell repository of National Center for Cell Science (NCCS), Pune, India, and maintained in DMEM (HiMedia, India) with 10% FBS (HiMedia, India). HEK293T cells were transfected with the pLKO.1-FRG1sh vector (Sigma, USA) for FRG1 knockdown and with the pLKO.1-scrambled sequence vector to get the corresponding control. Ice-cold PBS was used to wash the cells, and then cells were lysed in ice-cold 1X RIPA lysis buffer (Thermo Scientific, USA) supplemented with phosphatase inhibitor cocktail (Abcam, UK). We used BCA protein estimation kit (Thermo Scientific, USA) for protein quantification. Twenty micrograms of protein sample was mixed with an equal volume of 4 × Laemmili buffer and boiled at 95 °C for 5 min. The protein lysates were separated on SDS/PAGE (12% gel). The separated proteins were transferred to a PVDF membrane (Merck Millipore, USA), and the blot was treated with a blocking solution (5% BSA) for an hour. Blots were washed and treated with FRG1 primary antibody (Abcam, UK, 1:10,000 dilution), followed by anti-mouse IgG secondary antibody (AbGenex, India, 1:20,000 dilution). SuperSignal® West Femto reagent (Thermo Scientific, USA) was used to detect the chemiluminescence signal in ChemiDoc XRS + (Bio-Rad, USA). GAPDH (AbGenex, India) was used to normalize the sample band intensities.

### qRT-PCR

Total RNA was extracted using the RNeasy Mini kit (Qiagen, Germany). RNA was quantified using the NanoDrop 2000 spectrophotometer (Thermo Scientific, USA). RNA was converted into cDNA using oligo dT primer and random hexamer (in 1:3 ratio) (Verso cDNA Synthesis Kit). RT-PCR primers were designed for FRG1, HPF1, RPL34, and EXOSC9 using Primer-BLAST^23^ (Supplementary Table S1). SYBR™ Green PCR Master Mix (Applied Biosystems™, Thermo Scientific, USA) was used to perform qRT-PCR in QuantStudio™ 3 Real-Time PCR System (Applied Biosystems™, Thermo Scientific, USA). The experiment was performed in triplicate for each sample and GAPDH was used as an internal control.

### Statistics

For multigene model-based OS analysis multivariate Cox regression analysis was performed using SPSS (version 26)^24^. Risk score (generated via multigene model) based OS analysis was performed using Kaplan Meir analysis in SPSS. A log-rank test was used to find the statistical significance of the difference in survival between the groups. The prognostic value of the risk score was measured using a time-dependent receiver operating characteristic (ROC) curve in SPSS. Mean values were compared using Student's t-test (two-tailed, unpaired). For all the tests performed, a p-value ≤ 0.05 was considered significant.

## Results

### Effect of FRG1 alone on survival in different cancer types

**Figure 2:**
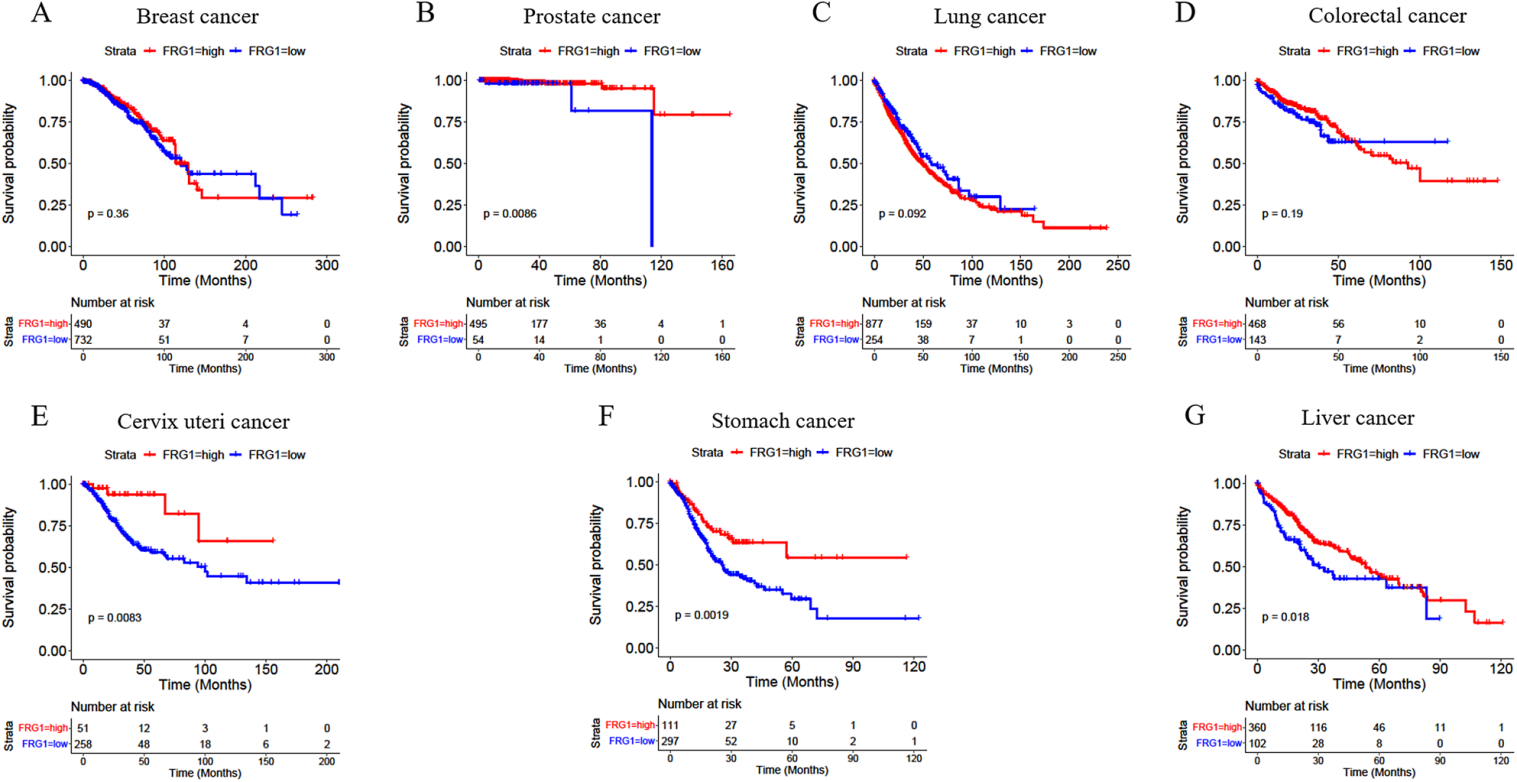
Kaplan–Meier plots showing overall survival with respect to FRG1 mRNA expression levels in different cancer types. Survival curves are shown for (**A**) Breast cancer (logrank P = 0.36), (**B**) Prostate cancer (logrank P = 0.0086), (**C**) Lung cancer (logrank P = 0.092) (**D**) Colorectal cancer (logrank P = 0.19) (**E**) Cervix uteri cancer (logrank P = 0.0083), (**F**) Stomach cancer (logrank P = 0.0019) and (**G**) Liver cancer (logrank P = 0.018). The X-axis represents the number of patients at risk at specific time (in months) and Y-axis shows the probability of survival. Red lines indicate FRG1-high expression group and blue lines indicate FRG1-low expression group divided based on logrank P test.

Kaplan-Meir survival analysis was performed to determine the effect of FRG1 mRNA expression on the OS across the seven most frequent cancer types. There was a highly significant difference in the survival probability between high and low FRG1 expression groups in cervix, stomach, and prostate cancers (Fig. 2). In liver cancer, the difference in survivability was marginally significant. Although the trend was there yet, the difference was not significant in breast, lung, and colorectal cancers. We used KM plotter data (available for breast cancer, lung adenocarcinoma, cervical squamous cell carcinoma, stomach adenocarcinoma, liver hepatocellular carcinoma, and rectum adenocarcinoma) for the validation. We observed a similar trend of the effect of FRG1 mRNA expression level on the OS as in the first set (Supplementary Fig. S2). Overall, the data suggest that FRG1 affects the survival in cancers but the extent of the effect is tissue specific. Analysis of FRG1 expression alone may not be enough to explain the contribution of other genes, which are affected by FRG1 directly or indirectly. Therefore, we did multigene model-based analysis in breast, lung, colorectal, and liver cancers to get a clear idea about the effect of FRG1 mRNA expression on OS.

### High FRG1 expression is associated with a good prognosis in the multigene model

To determine the contribution of FRG1 on survival, the effect correlated genes was neutralized using the multivariate Cox regression model. Sub-sections below describe the multigene model for each cancer type.

### Effect of FRG1 and correlated genes on survival in breast cancer

In breast carcinoma, initially, we entered the top 20 FRG1 correlated genes (r_s_ ≥ 0.353) (Supplementary Table S2) to generate the multivariate cox regression model in the TCGA-BRCA (The Cancer Genome Atlas Breast Invasive Carcinoma) dataset. Sequentially the least correlated genes were removed from the model until the FRG1 showed a maximum level of significant association with survival (Table 1). The hazard ratio of FRG1 was 0.133 (95% CI 0.029–0.599, p = 0.009) for breast cancer patient’s death.

**Table 1:** Covariates present in multivariate Cox regression model in breast cancer patients.

Genes	B	Sig	Exp(B), 95.0% CI for Exp(B)
HPF1	1.233	0.134	3.433 (0.683,17.259)
ING2	− 1.865	0.003	0.155 (0.045,0.535)
UFSP2	2.672	0	14.467 (3.49,59.965)
PFDN5	− 1.501	0.089	0.223 (0.04,1.256)
EXOSC9	− 1.376	0.08	0.253 (0.054,1.177)
SARNP	0.939	0.049	2.557 (1.005,6.504)
SRP19	− 0.34	0.688	0.712 (0.135,3.743)
RPS3A	− 0.76	0.39	0.468 (0.083,2.647)
NDUFC1	− 0.472	0.514	0.624 (0.151,2.577)
NACA	0.959	0.302	2.609 (0.422,16.14)
RWDD4	− 0.247	0.774	0.781 (0.145,4.201)
NSA2	− 0.578	0.389	0.561 (0.15,2.092)
TBCA	1.132	0.136	3.102 (0.7,13.75)
MRPS18C	0.698	0.401	2.01 (0.395,10.239)
TRIM56	− 0.226	0.663	0.797 (0.288,2.205)
TTC1	− 0.236	0.792	0.79 (0.137,4.549)
PLRG1	0.758	0.368	2.134 (0.409,11.134)
MRPL1	0.536	0.498	1.71 (0.362,8.069)
RPL34	0.588	0.464	1.8 (0.373,8.688)
FRG1	− 2.021	0.009	0.133 (0.029,0.599)
Age (months)	0.003	0	1.003 (1.002,1.004)

**Figure 3:**
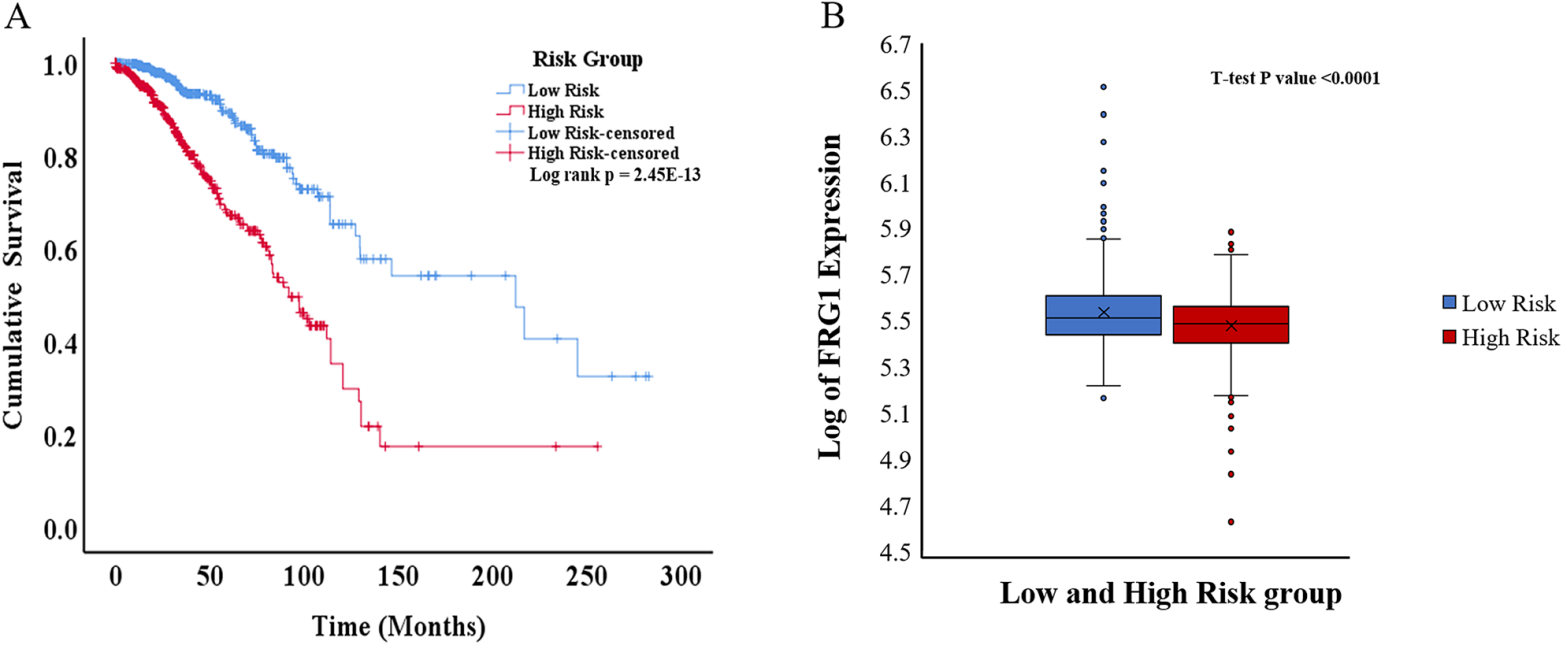
Kaplan–Meier plot and box plot of breast cancer patients risk groups based on the multigene model. (**A**) KM plot showing overall survival in low-risk and high-risk patient groups (Log-rank test p-value = 2.45E−13). The blue line shows the low-risk group, and the red line shows the high-risk group. (**B**) Box plot showing log of FRG1 expression level in low and high-risk groups. The Y-axis represents the log of FRG1 expression, and the X-axis shows the group.

To analyze the combined effect of FRG1 and the correlated genes (genes present in the final model) on the OS, for each breast cancer patient risk score was calculated. The patients were stratified into low-risk (n = 612) and high-risk (n = 611) groups based on the median risk score value. A significant (p = 2.45E-13) difference in OS was observed between the groups (Fig. 3A). The AUC (area under the ROC Curve) for this risk model was 0.645 (Supplementary Fig. S3). In Time-dependent ROC curve analysis the value of AUC above 0.5 indicates a good prognostic performance based on risk factor in predicting the overall survival. There was significantly higher (p < 0.0001) FRG1 mRNA expression in the low-risk group compared to the high-risk group (Fig. 3B).

### Effect of FRG1 and correlated genes on survival in Lung cancer

The top 20 FRG1 correlated genes (r_s_ ≥ 0.535) (Supplementary Table S2) and FRG1 were added in multivariate cox regression model using TCGA-MESO (The Cancer Genome Atlas Mesothelioma), TCGA-LUAD (The Cancer Genome Atlas Lung Adenocarcinoma) and TCGA-LUSC (The Cancer Genome Atlas Lung Squamous Cell Carcinoma) datasets. To investigate the prognostic effect of FRG1 on lung carcinoma patients, we applied the same strategy as described above. The final model had 17 genes where the hazard ratio of FRG1 was 0.235 (95% CI 0.074–0.742, p = 0.014) for lung cancer patient’s death (Table 2).

**Table 2:** Covariates present in multivariate Cox regression model in lung cancer patients.

Genes	B	Sig	Exp(B), 95.0% CI for Exp(B)
HPF1	0.801	0.09	2.227 (0.882,5.621)
MRPS18C	0.449	0.373	1.566 (0.584,4.2)
ANAPC10	− 1.668	0.008	0.189 (0.055,0.652)
LSM6	0.725	0.149	2.064 (0.771,5.525)
ATP5PO	− 0.004	0.993	0.996 (0.425,2.338)
UBE2B	0.018	0.966	1.019 (0.432,2.403)
THOC7	− 0.65	0.229	0.522 (0.181,1.504)
NDUFS4	0.347	0.362	1.416 (0.671,2.988)
RWDD4	0.331	0.53	1.393 (0.495,3.917)
COX7B	− 0.295	0.477	0.744 (0.33,1.681)
GSTO1	0.368	0.191	1.444 (0.833,2.505)
RPL24	0.143	0.691	1.154 (0.57,2.335)
UBE2D3	0.951	0.113	2.587 (0.799,8.377)
UXT	− 0.246	0.617	0.782 (0.299,2.048)
LSM3	0.791	0.124	2.206 (0.805,6.041)
RPL34	− 0.508	0.157	0.602 (0.298,1.216)
SHANK2	0.221	0.048	1.247 (1.002,1.552)
FRG1	− 1.448	0.014	0.235 (0.074,0.742)
Age (months)	0.001	0.025	1.001 (1,1.002)

**Figure 4:**
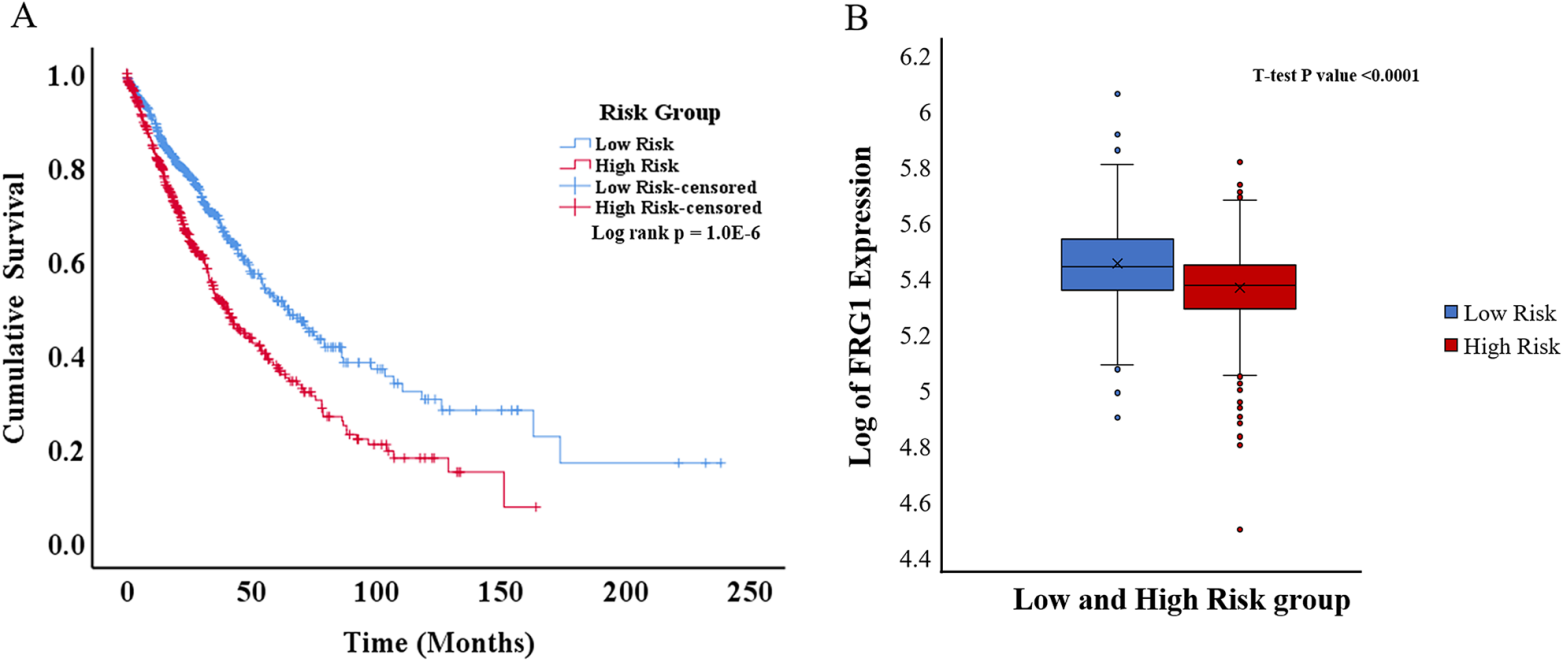
Kaplan–Meier plot and box plot of lung cancer patients risk groups based on the multigene model. (**A**) KM plot showing overall survival in low-risk and high-risk patient groups (Log-rank test p-value = 1.0E−6). Blue line shows the low-risk group and the red line shows the high-risk group. (**B**) Box plot showing log of FRG1 expression level in low and high-risk groups. The Y-axis represents the log of FRG1 expression and the X-axis shows the group.

All the patients were stratified into low-risk (n = 559) and high-risk (n = 572) groups based on the median value of the risk score. The AUC for this risk model was 0.569 (Supplementary Fig. S3). A significant difference (p = 1.0E−6) in OS was observed between the groups (Fig. 4A). There was significantly (p < 0.0001) high FRG1 expression in the low-risk group compared to the high-risk group (Fig. 4B).

### FRG1 and correlated genes do not predict survival in the colorectal cancer

To investigate the prognostic effect of FRG1 using colorectal cancer TCGA-READ (The Cancer Genome Atlas Rectum Adenocarcinoma) and TCGA-COAD (The Cancer Genome Atlas Colon Adenocarcinoma) datasets, the top 20 FRG1 correlated genes (Supplementary Table S2) were added (r_s_ ≥ 0.964) in the multivariate cox regression model (Supplementary Table S3). Models with all the 20 genes and any other combination of genes didn’t show significant effect of FRG1 on the OS of the colorectal cancer patients. The hazard ratio of FRG1 was 0.478 (95% CI 0.081–2.824, p = 0.415) for colorectal cancer patient’s death.

**Figure 5:**
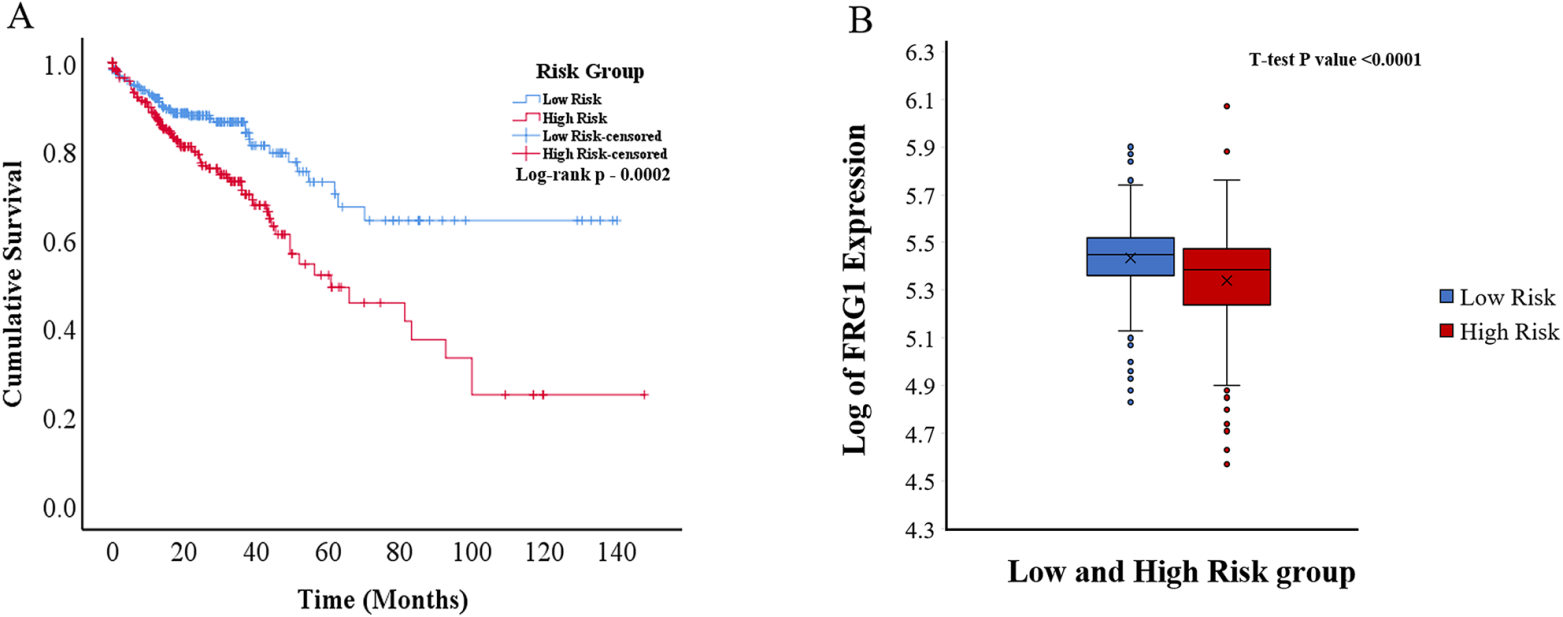
Kaplan–Meier plot and box plot of colorectal cancer patients risk groups based on the multigene model. (**A**) KM plot showing overall survival in low-risk and high-risk patient groups (Log-rank test p-value = 0.0002). The blue line shows the low-risk group and the red line shows the high-risk group. (**B**) Box plot showing log of FRG1 expression level in low and high-risk groups. The Y-axis represents the log of FRG1 expression and the X-axis shows the group.

Next, to determine the effect in the multigene model, the patients were divided into the low-risk (n = 306) and high-risk groups (n = 305), based on the median risk score. The AUC for this risk model was 0.604 (Supplementary Fig. S3). A significant (p = 0.0001) difference in OS was observed between the two groups (Fig. 5A). Comparison of FRG1 mRNA expression between the high-risk and low-risk groups showed significantly (p < 0.0001) higher expression in the low-risk group (Fig. 5B).

### Effect of FRG1 and correlated genes on survival in liver cancer

The top 20 FRG1 correlated genes (Supplementary Table S2), with r_s_ cutoff ≥ 0.539, were used to generate the multivariate cox regression model using TCGA-LIHC (The Cancer Genome Atlas Liver Hepatocellular Carcinoma) and TCGA-CHOL (The Cancer Genome Atlas Cholangiocarcinoma) datasets. The final model had 16 genes (Table 3) where the hazard ratio of FRG1 was 0.18 (95% CI 0.034–0.948, p = 0.043) for liver cancer patient’s death.

**Table 3:** Covariates present in multivariate Cox regression model in liver cancer patients.

Genes	B	Sig	Exp(B), 95.0% CI for Exp(B)
HPF1	0.791	0.238	2.206 (0.593,8.209)
POMP	0.885	0.174	2.424 (0.676,8.691)
UXT	− 0.222	0.772	0.801 (0.179,3.591)
RREB1	− 1.001	0.089	0.367 (0.116,1.164)
LMTK2	− 0.551	0.289	0.576 (0.208,1.596)
NDUFC1	− 1.594	0.009	0.203 (0.061,0.676)
EP300	0.786	0.335	2.195 (0.445,10.844)
NCOA2	− 0.613	0.146	0.542 (0.237,1.239)
MRPL54	− 1.53	0.009	0.217 (0.069,0.683)
KMT2C	− 0.615	0.259	0.541 (0.186,1.574)
PRR14L	− 0.073	0.914	0.93 (0.251,3.449)
UFSP2	1.583	0.022	4.871 (1.254,18.923)
HACD2	0.919	0.033	2.507 (1.075,5.846)
CELF1	0.741	0.421	2.097 (0.346,12.73)
NCOA6	− 0.044	0.954	0.957 (0.216,4.245)
NDUFS5	1.581	0.004	4.86 (1.681,14.049)
FRG1	− 1.716	0.043	0.18 (0.034,0.948)
Age (Months)	0.001	0.055	1.001 (1,1.002)

**Figure 6:**
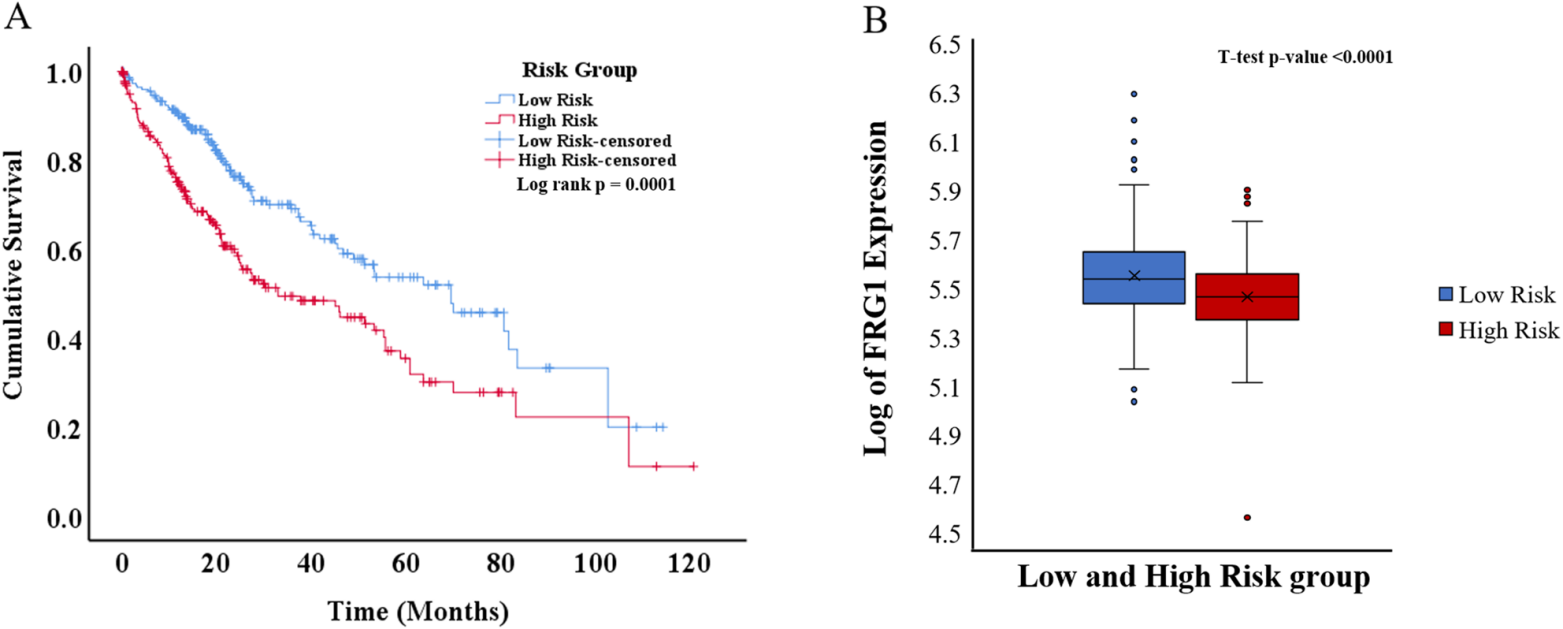
Kaplan–Meier plot and box plot of liver cancer patients risk groups based on the multigene model. (**A**) KM plot showing overall survival in low-risk and high-risk patient groups (Log-rank test p-value = 0.0001). The blue line shows the low-risk group, and the red line shows the high-risk group. (**B**) Box plot showing the log of FRG1 expression level in low and high-risk groups. The Y-axis represents the log of FRG1 expression, and the X-axis shows the group.

Next, to determine the effect in the multigene model, the patients were divided into the low-risk group (n = 231) and high-risk group (n = 231) based on the median risk score. The AUC for this risk model was 0.616 (Supplementary Fig. S3). A significant (p = 0.0001) difference in OS was observed between the two groups (Fig. 6A). Comparison of FRG1 mRNA expression between the high-risk group and low-risk group (Fig. 6B) showed significantly (p < 0.0001) higher expression in the low-risk group.

### FRG1 knockdown in HEK293T reduces expression of HPF1, RPL34, and EXOSC9

**Figure 7:**
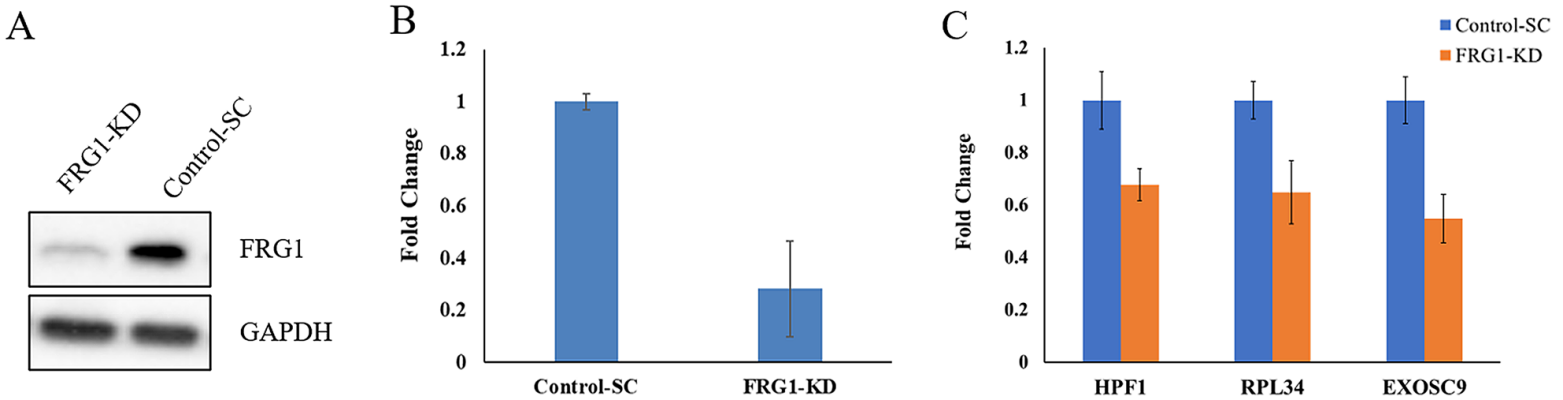
Effect of FRG1 expression on other genes. (**A**) Western blot showing FRG1 expression in HEK293T cells with FRG1 knock down (FRG1-KD) and respective control (Control-SC). (**B**) The bar graph shows the levels of FRG1 mRNA in HEK293T cells with FRG1 knock down (FRG1-KD) and respective control (Control-SC) (n = 3). (**C**) The bar graph shows the change in mRNA expression of HPF1, RPL34, and EXOSC9 in HEK293T cells with FRG1 knockdown (FRG1-KD) compared to the control (Control-SC) (n = 3). Y-axis shows fold change in expression using GAPDH as an internal control.

To validate the knockdown of FRG1 in HEK239T cells, we performed Western blot and qRT-PCR analysis, which confirmed a significant decrease in the FRG1 expression (Fig. 7). From the top 20 genes correlated with FRG1 across cancer types, we found that three genes (HPF1, RPL34, and EXOSC9) were common. We hypothesized that these genes could be part of pathway/pathways in which FRG1 has a role and could affect their expression. To validate this, the expression level of these three genes was analyzed in response to FRG1 depletion in the HEK293T cell line by quantitative real-time PCR. We observed that knockdown of FRG1 led to a significant decrease in expression of HPF1 (0.68-fold, p-value = 0.011), RPL34 (0.65-fold, p-value = 0.025) and EXOSC9 (0.54-fold, p-value = 0.012) (Fig. 7). These findings suggest the effect of FRG1 in transcriptional regulation of HPF1, RPL34, and EXOSC9, which could be direct or indirect.

### FRG1 may have role in multiple pathways

**Figure 8:**
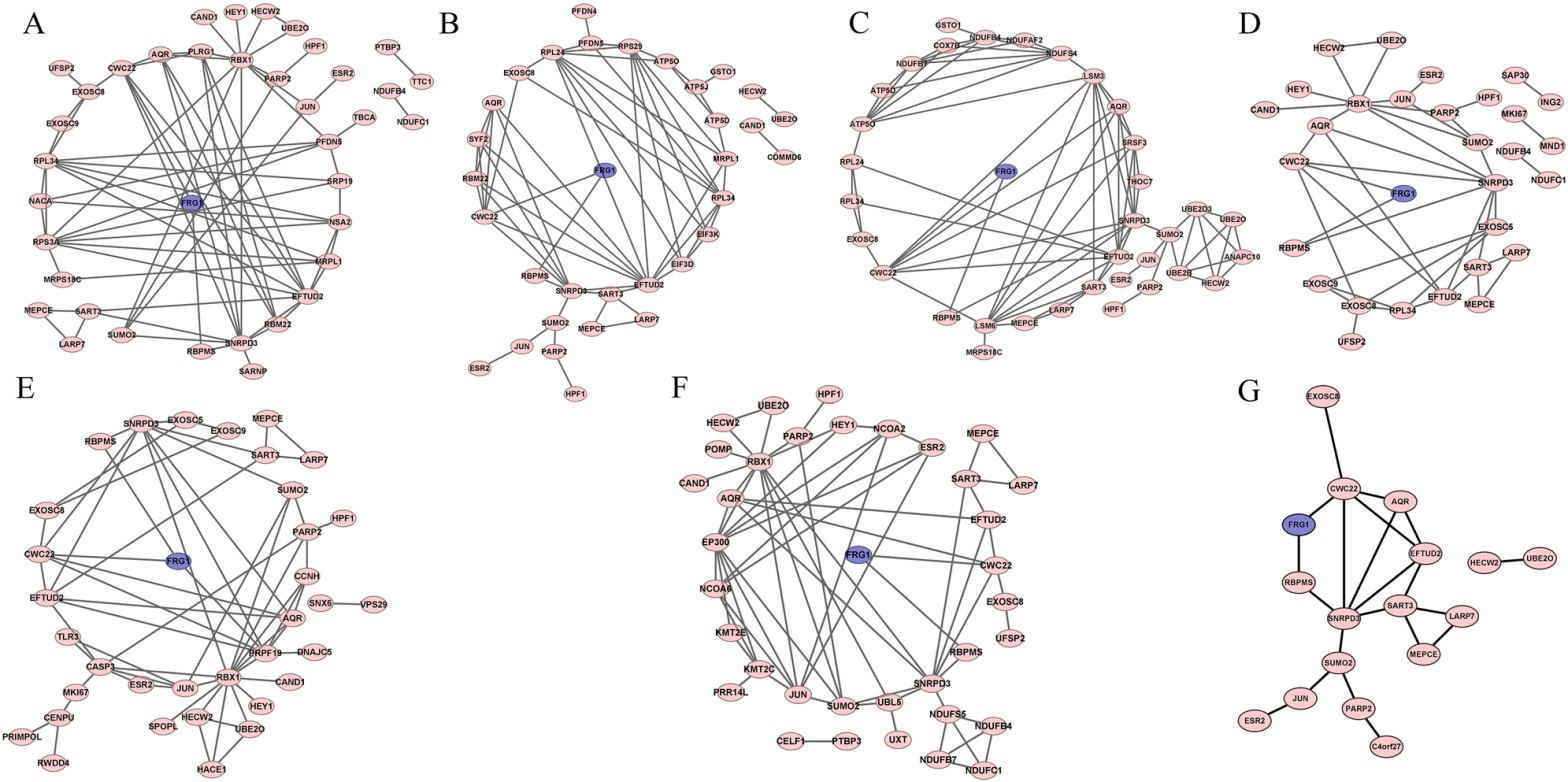
Co-expression and protein–protein interaction network analysis of FRG1 in different cancer types. Networks show FRG1 in blue at the center, and other genes with pink for (**A**) Breast Cancer (node = 38, edge = 72), (**B**) Prostate cancer (node = 33, edge = 62), (**C**) Lung Cancer (node = 35, edge = 78, (**D**) Cervix-Uteri cancer (node = 30, edge = 42), (**E**) Stomach cancer (node = 35, edge = 61), and (**F**) Liver Cancer (node = 36, edge = 64). (**G**) Common network across cancer types (node = 17, edge = 21). Nodes represent the number of genes and edges define interaction between genes.

**Figure 9:**
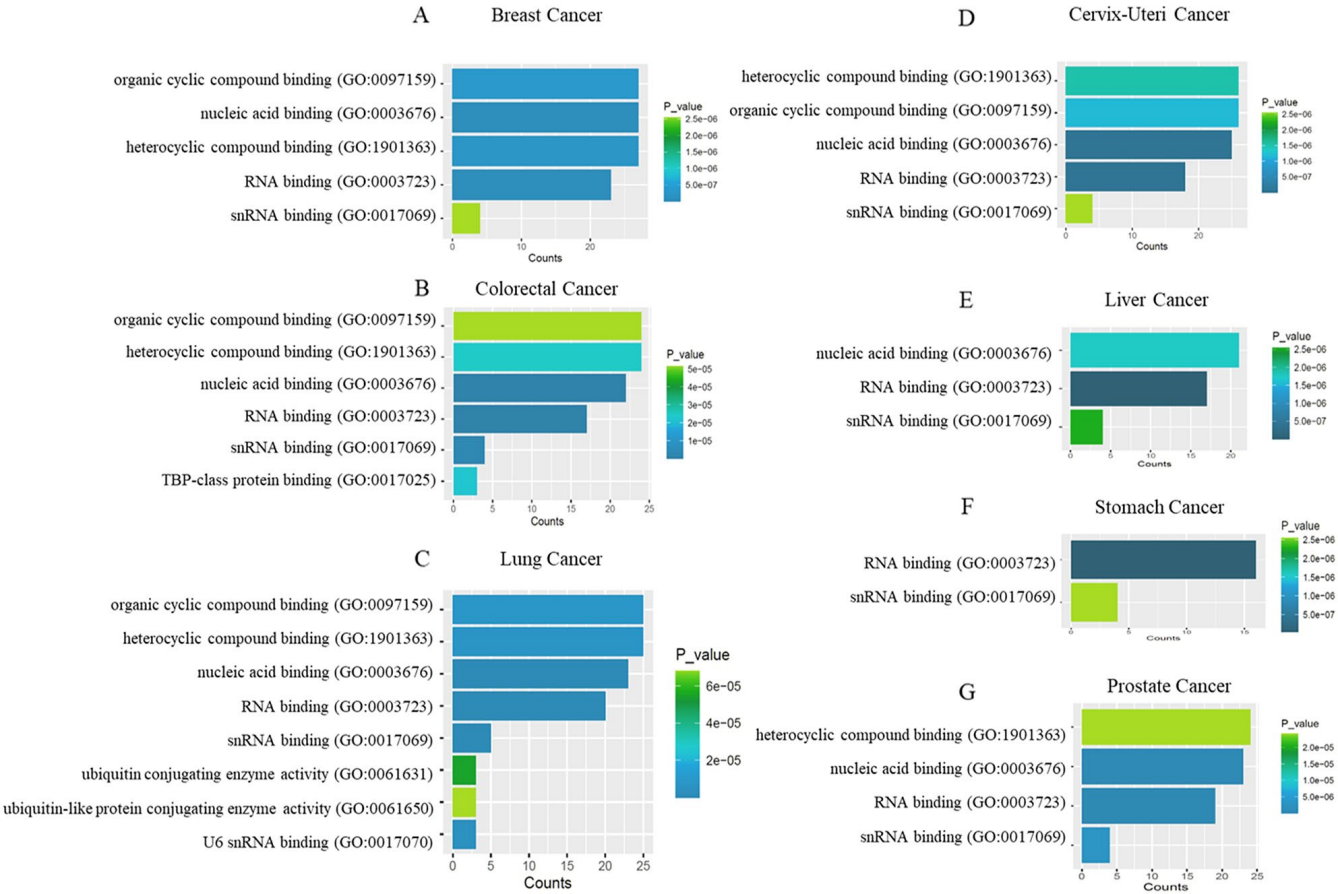
Significantly enriched GO terms for molecular function using FRG1 and correlated genes. (**A**) Breast cancer, (**B**) Colorectal cancer, (**C**) Lung cancer, (**D**) Cervix uteri cancer, (**E**) Liver cancer, (**F**) Stomach cancer, and (**G**) Prostate cancer. The X-axis represents gene counts and Y-axis shows the GO term/molecular function.

To figure out the pathway/s where FRG1 may have a role we used genes that correlate with FRG1 expression and the genes that interact with FRG1 (HIPPIE database) as input in the STRING database. Individual networks for each cancer type are shown in Fig. 8. After that all the networks were merged and the intersection was obtained using the Merge tool of Cytoscape, giving us the most common pathway (Fig. 8). The merged pathway had 17 nodes (MEPCE, LARP7, SUMO2, UBE2O, HECW2, RBPMS, JUN, ESR2, SART3, EXOSC8, FRG1, PARP2, C4orf27 (HPF1), EFTUD2, SNRPD3, CWC22, and AQR) and 21 edges. Functional enrichment analysis showed GO terms RNA binding, snRNA binding, and nucleic acid binding to be most frequent in molecular functional (Fig. 9). In biological processes (Supplementary Fig. S4) we observed GO terms such as metabolic process, RNA metabolic process, and mRNA metabolic process to be the most frequent. We identified Ribosome KEGG pathway to be common in different cancer types (Supplementary Table S4). Other RNA related pathways, namely spliceosome and RNA degradation pathways were also identified in lung cancer.

## Discussion

FRG1 protein is part of human spliceosomal complex C^25^. Earlier studies primarily focused on the role of FRG1 in FSHD. However, a few studies have demonstrated the role of FRG1 in tooth germ development and angiogenesis^6,26^. Our previous research showed reduction in FRG1 protein expression in gastric cancer, colon cancer, and oral cavity cancer tissues by IHC analysis. Change in FRG1 mRNA expression affected migration and angiogenic potential of HEK293T and HUVECs, respectively. FRG1 expression perturbation in HEK293T cell affected G-CSF (granulocyte-colony stimulating factor) and MMP10 (matrix metalloproteinase 10) levels^6^. Both G-CSF and MMP10 play an important role in cell migration and tumor progression properties of cancer^27,28^. Our another study clearly showed the protective role of FRG1 in prostate cancer^7^. FRG1 expression was reduced in prostate tumor tissues compared to normal tissue. Depletion of FRG1 led to increased tumorigenic properties in prostate cancer cell lines and activation of p38 MAPK (mitogen-activated protein kinase) signaling. FRG1 expression affected levels of GM-CSF (Granulocyte Macrophage colony stimulating factor), PLGF (Placental Growth Factor), PDGFA (Platelet Derived Growth Factor A) and CXCL1 (Chemokine (C-X-C motif) ligand 1), which are well known for their effect on tumor progression, chemotaxis, migration and invasion^29–34^. Being part of the spliceosomal C complex, FRG1’s downregulation might lead to instability and disruption in downstream processes affecting the normal mRNA levels. In concordance, recent studies have shown that the expression of splicing factors is frequently deregulated in different cancer types^35^.

Role of FRG1 in survival of cancer patients is not clearly understood. There aren’t many studies focusing on FRG1, hence we wanted to perform a comprehensive study in multiple cancer types to elucidate a concreate role of FRG1 in predicating the OS of cancer patients. We first elucidated the role of FRG1 alone in the OS in multiple cancer types. High FRG1 mRNA expression correlated with better survival in the cervix and gastric cancer patients. In cancer types such as breast, lung, and liver, the difference in FRG1 expression level did not affect OS significantly. We observed that the patients with low FRG1 mRNA expression were more frequent in the cervix and gastric cancers. On the contrary, just the opposite trend was observed in liver, colorectal, lung, and prostate cancers. In breast cancer, distribution was approximately equal. Expression of genes can correlate if one of them regulates the transcription of another, directly or indirectly. Upstream regulator genes may have mutation/s, resulting in the masking of independent effects of mutation/s in the downstream target. We used multigene models to nullify the influence of other genes on OS that correlate with FRG1. As expected, we observed a clear effect of FRG1 expression in breast, lung, and liver cancers also after multivariate cox regression analysis.

Segregation of the patients based on the risk score (calculated based on the multigene model) showed that low-risk patients had better OS than high-risk patients. We also observed that low-risk patients had high FRG1 levels, which confirms the role of FRG1 mRNA expression in survival. The earlier studies support our observation directly, where increased FRG1 expression affected in-vitro cell migration, invasion, and angiogenesis inversely^6^.

**Figure 10:**
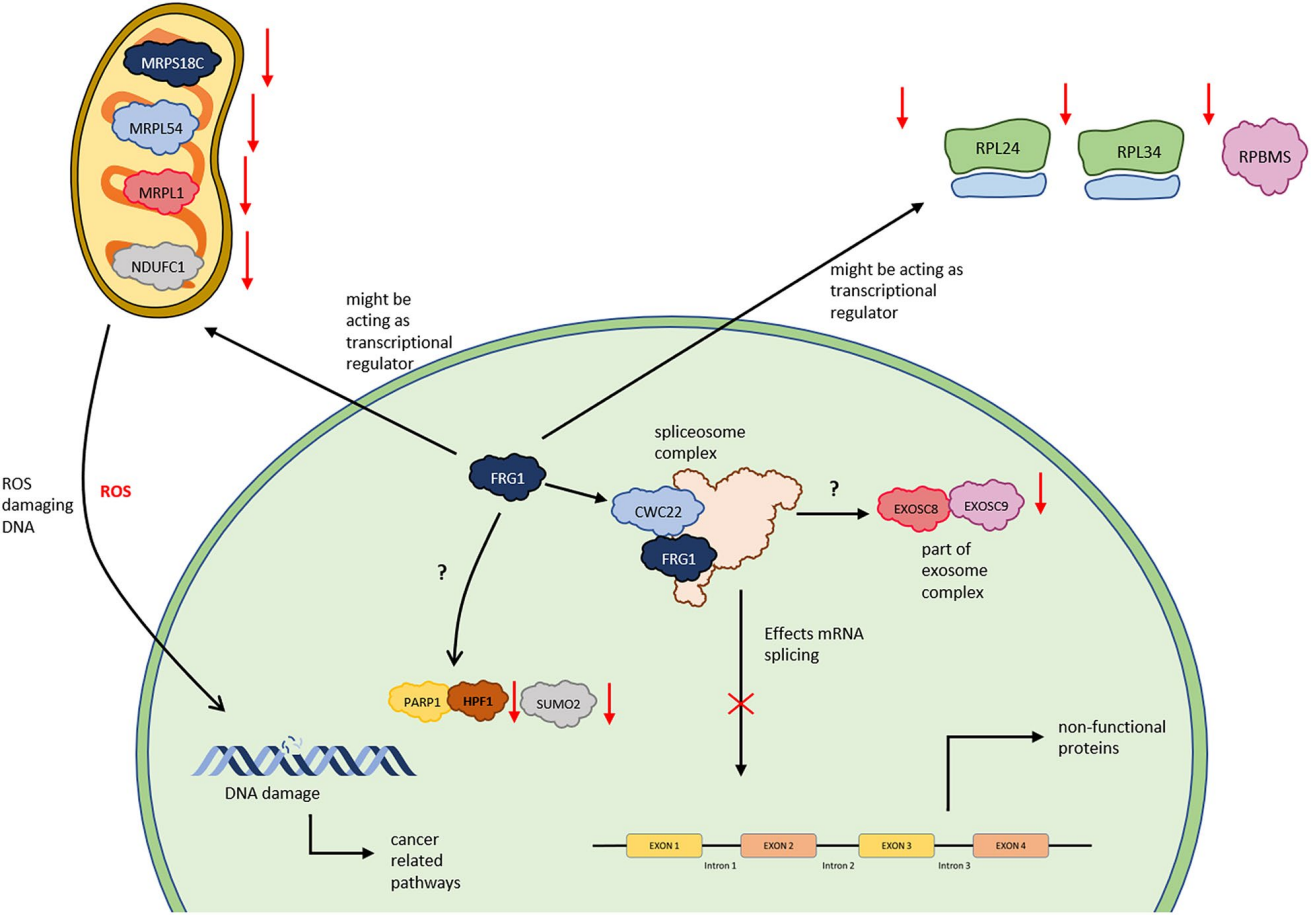
Hypothetical model showing functions of FRG1, based upon expression correlation and protein–protein interactions. Red downward arrows indicate the downregulation of expression, and the red cross shows inhibition.

To further elucidate the molecular mechanism of the role of FRG1 in cells, we generated pathways. Our final model (Fig. 10) shows four types of functions where FRG1 might be involved, namely pre-mRNA processing (CWC22), mitochondrial functioning (MRPS18C, MRPL1, MRPL54, and NDUFC1), ribosomal functioning (RPL34, RPL24), and in DNA damage/repair pathway (HPF1, PARP1, SUMO2). FRG1 with CWC22 interact^36^, and they both are also part of the spliceosomal C complex^25^. Deregulation of these genes may have a direct effect on spliceosome complex functioning. Previous literature has shown the importance of CWC22 in pre-mRNA splicing^37^. CWC22 expression levels were associated with colon cancer and its silencing led to increased p53 levels^38,39^. SNRPD3 is also part of the spliceosome complex^40^. It has been found to have a regulatory effect on p53 expression in non-small cell lung cancer. It also has a role in triple-negative breast cancer cell proliferation^38,41^. In our model we found EXOSC9 to be highly correlated with FRG1 in multiple cancer types. Protein–protein interaction between FRG1 and EXOSC8 has been observed in previous studies^42^. EXOSC8 and EXOSC9 (both present in our model) are non-catalytic parts of the RNA exosome complex^43^. EXOSC8 and EXOSC9 are associated with many diseases^44,45^, but their role in cancer has recently been uncovered. EXOSC8 promoted tumor and cancer cell growth in colorectal carcinoma^46^. Reduction in EXOSC9 was associated with reducing of p-body formation in cancer cells^47^. From all these studies, we can infer that FRG1 along with EXOSC8 and EXOSC9 might play a major role in controlling RNA processing and, its depletion can affect functional RNAs. Our Functional enrichment analysis results also suggest FRG1 may be involved in RNA related biological processes and molecular functions.

Another very interesting observation was the mitochondria-related genes in our model. Mitochondrial ribosomal proteins (MRPS18C, MRPL1, and MRPL54) and NDUFC1, which is a component of the mitochondrial complex 1, are related to FRG1. MRPS18C is downregulated in esophageal cancer^48^. MRPL1 is a part of the gene signature for low-grade gliomas prognosis^49^. In malignant mesothelioma (MM) and lung cancer MRPL1 was mutated^50^. Similarly, in HCC, high expression of MRPL54 was associated with better survival^51^. NDUFC1 may affect the production of ROS, which has been observed in many cancer types^52^. Similarly, RPL24 and RPL34 that are part of the cytoplasmic ribosomal complex, can affect protein production. Alteration in RPL34 expression affects non-small cell lung cancer cell proliferation^53,54^. Depletion of RPL24 inhibits cancer cell growth, which makes RPL24 a potential therapeutic target^55^. Another gene in our model, RBPMS interacts with FRG1 at protein level^56^. RBPMS has been shown as a coactivator of transcriptional activity of many genes^57^. Multiple myeloma shows drug resistance when RBPMS is silenced^58^. These observations suggest that FRG1 might control the protein synthesis as well.

FRG1 is also related to the DNA repair pathway (HPF1, PARP1, and SUMO2). HPF1 protects the DNA from damage by limiting the hyper auto modification of PARP1 required for repair^59^. PARP2 shows a direct protein–protein interaction with FRG1, but its function is unknown. SUMO2, which plays an important role in post-translational modification and affects multiple cellular processes, including DNA repair and replication^60,61^, has also been implicated in cancers^62,63^. FRG1 may affect DNA repair by acting as a transcriptional regulator of these genes.

Overall, our analysis indicates two possibilities about FRG1’s role, first being a part of the spliceosome complex and the other is by acting as a transcriptional regulator of other genes involved in various functions. To check the latter possibility, we performed qRT-PCR and found that FRG1 knockdown led to a reduction in expression levels of HPF1, EXOSC9, and RPL34. Further in-depth experiments are needed to figure out the exact role of FRG1 in tumorigenesis via the first possibility. FRG1’s role as transcriptional activator or repressor may be assessed by identifying its direct binding to the promoter region of the putative target gene (EMSA, ChIP). Integrity of spliceosome complex and rate of transcription can be checked after knock out or knock down of FRG1. Immunoprecipitation assay can confirm the protien-protien interaction with spliceosome components. This study has additional limitations, such as more number of genes can be incorporated in our model. We chose the top seven cancer types with the highest incident rates; studies in other cancers can give a more in-depth understanding of the FRG1 pathway.

In conclusion, this study has clearly shown the role of FRG1 in predicting the survivability of cancer patients. The higher expression of the FRG1 gene has a protective effect. The use of the multigene models can be helpful in elucidating the effect of a specific gene in a biologically complex background.

## Supplementary Information

Supplementary Information 1.

Supplementary Information 2.

Supplementary Information 3.

Supplementary Information 4.

Supplementary Information 5.

Supplementary Information 6.
