# Supplementary material for: Role of FRG1 in predicting the overall survivability in cancers using multivariate based optimal model
Source: Sci Rep. 2021 Nov 18;11:22505. doi: 10.1038/s41598-021-01665-w (PMC8602605; doi:10.1038/s41598-021-01665-w)
Supplement: Supplementary file 1 — Supplementary Information 1. [file 41598_2021_1665_MOESM1_ESM.pdf]

# Role of FRG1 in predicting the overall survivability in cancers using multivariate based optimal model

Rehan Khan[1]

Ananya Palo[1]

Manjusha Dixit[1*]

**Affiliations:**

[1]School of Biological Sciences, National Institute of Science Education and Research, Bhubaneswar, HBNI, P.O. Jatni, Khurda 752050, Odisha, India

***Corresponding Author:** Manjusha Dixit

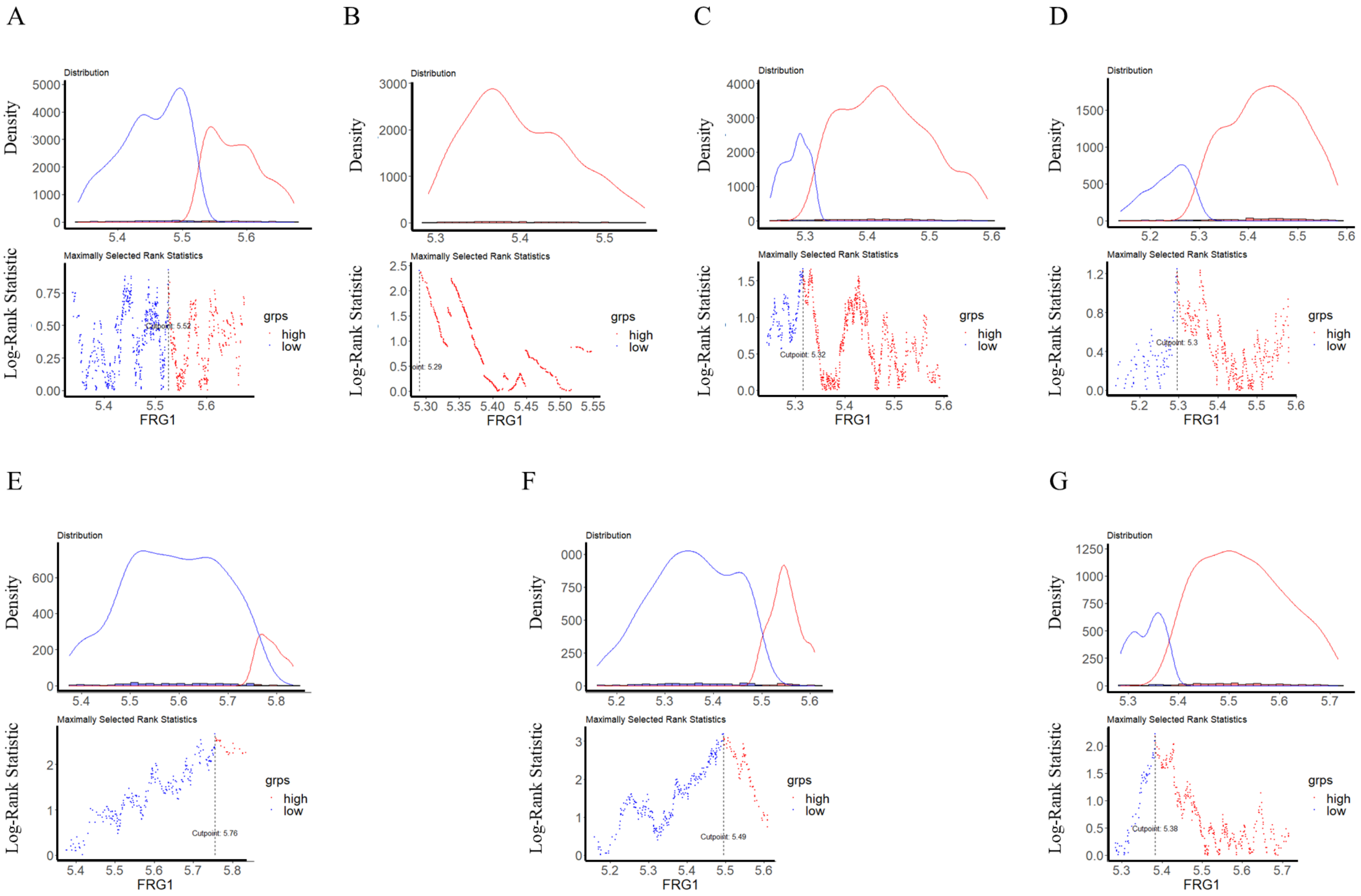


Supplementary Figure S1. Optimal cut-off points determination of FRG1 expression for KM plot in different cancer types. surv_cutpoint() function was used and plotted using plot() for (A) breast cancer, (B) Prostate cancer (C) lung cancer (D) Colorectal cancer (E) Cervix uteri cancer, (F) Stomach cancer and (G) Liver cancer.

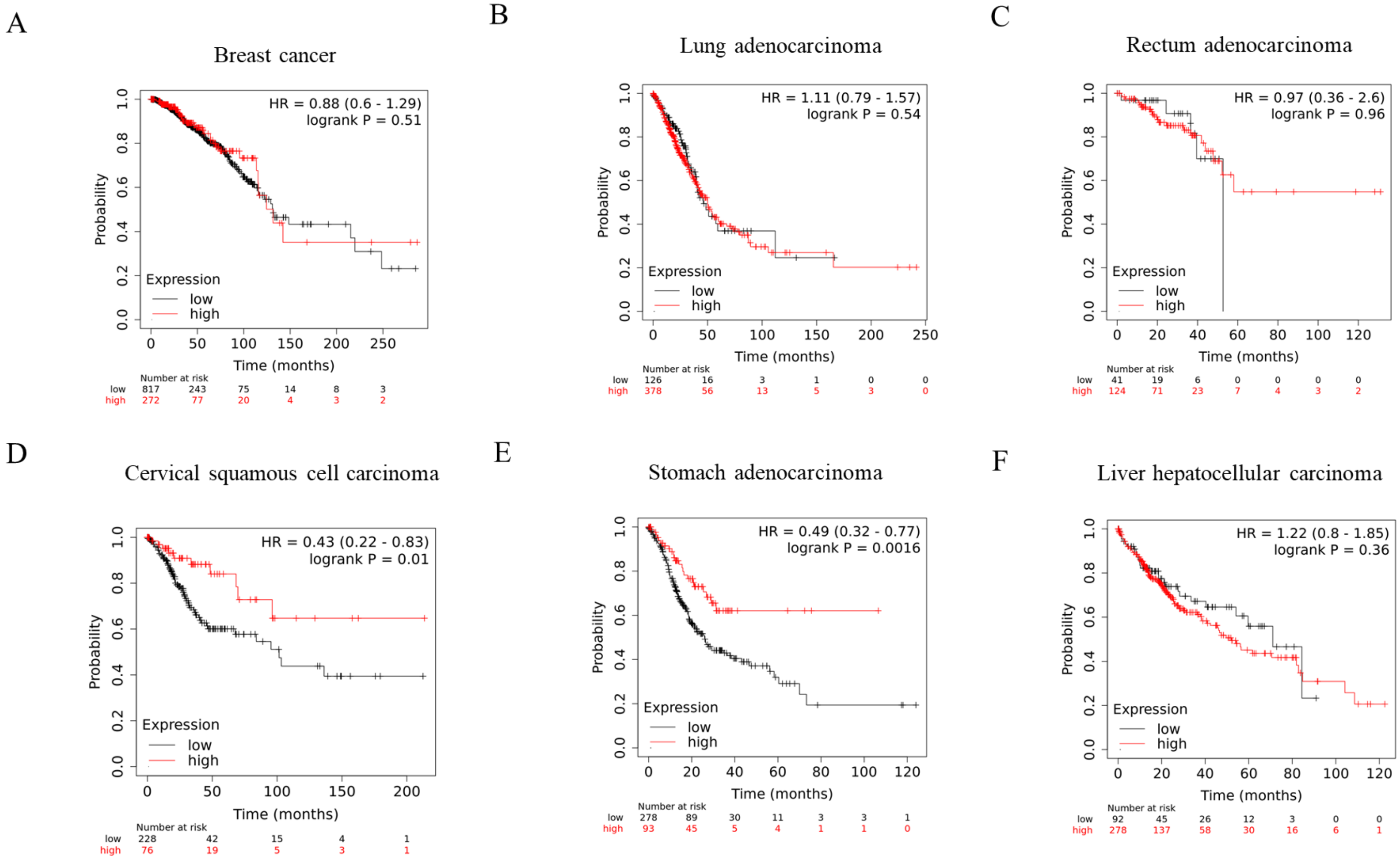


Supplementary Figure S2. Kaplan-Meier plots showing overall survival with respect to FRG1 expression levels in different cancer types analyzed in Kaplan-Meier Plotter using Pan-cancer RNA-seq. Survival curves are shown for (A) Breast cancer (logrank P = 0.51), (B) Lung adenocarcinoma (logrank P = 0.54), (C) Rectum adenocarcinoma (logrank P = 0.96) (D) Cervical squamous cell carcinoma (logrank P = 0.01) (E) Stomach adenocarcinoma (logrank P =0.0016) and, (F) Liver hepatocellular carcinoma (logrank P = 0.36). The X-axis represents the number of patients at risk at specific time (in months) and Y-axis shows the probability of survival. Red lines indicate FRG1-high expression group and black lines indicate FRG1-low expression group.

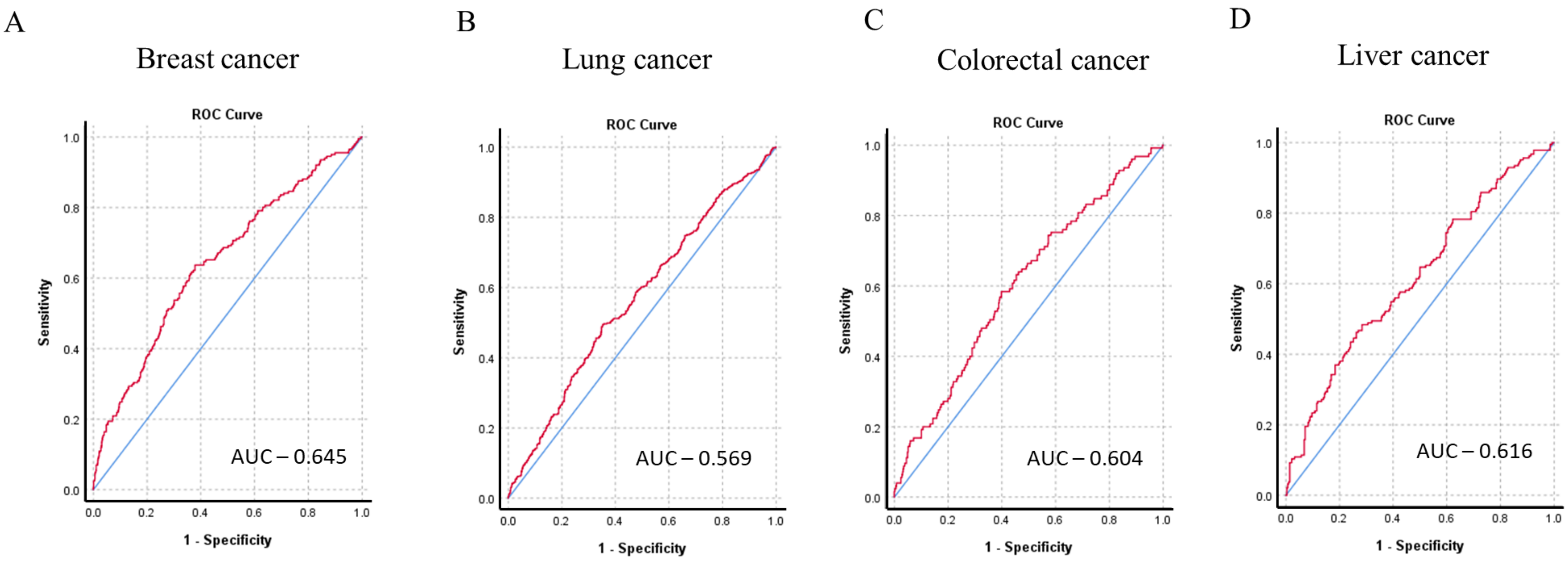


Supplementary Figure S3. The time-dependent receiver operating characteristic (ROC) curve for (A) Breast cancer, (B) Lung cancer (C) Colorectal cancer and, (D) Liver cancer.

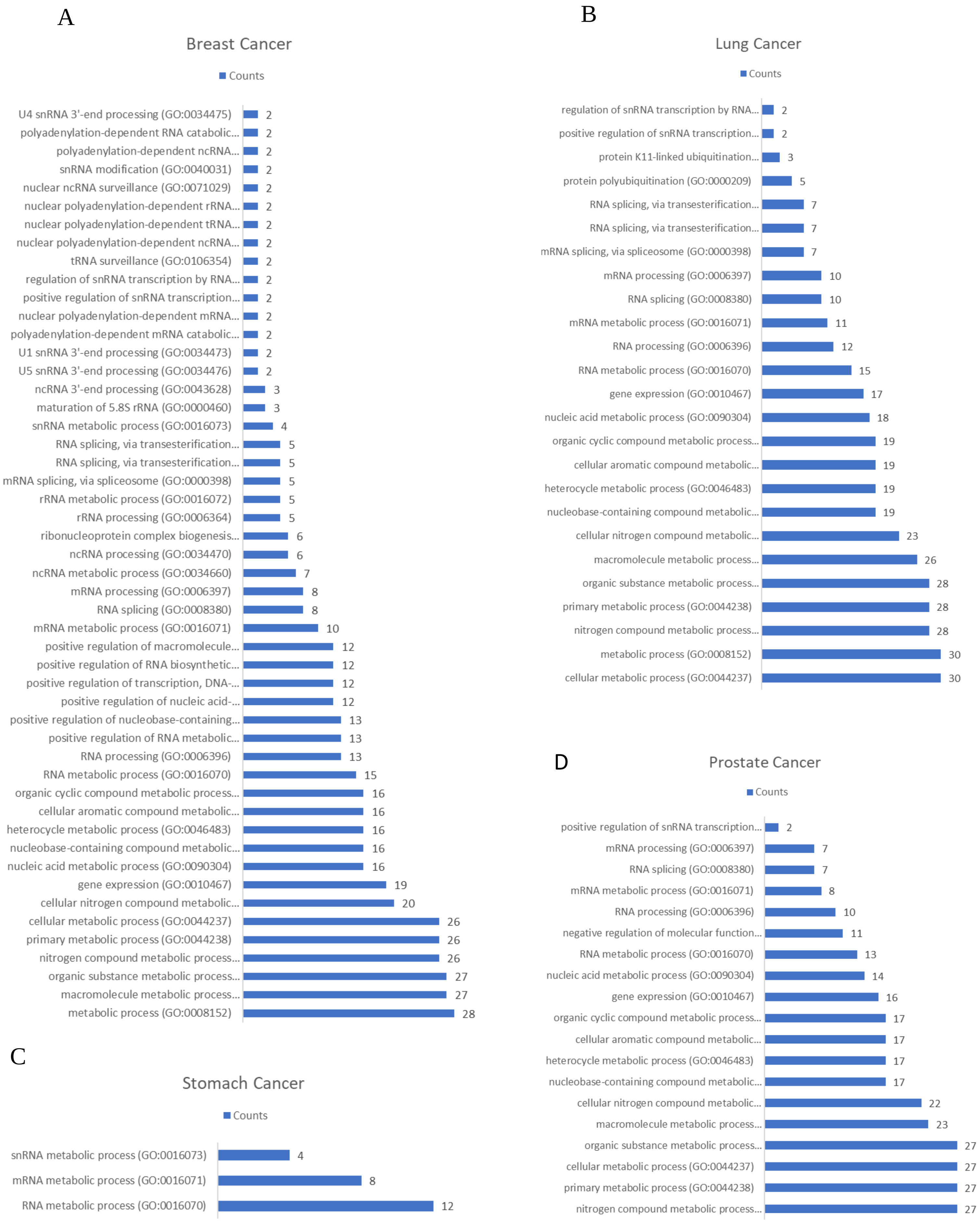
A
Breast Cancer
Counts
U4 snRNA 3'-end processing (GO:0034475) 2
polyadenylation-dependent RNA catabolic... 2
polyadenylation-dependent ncRNA... 2
snRNA modification (GO:0040031) 2
nuclear ncRNA surveillance (GO:0071029) 2
nuclear polyadenylation-dependent rRNA... 2
nuclear polyadenylation-dependent tRNA... 2
nuclear polyadenylation-dependent ncRNA... 2
tRNA surveillance (GO:0106354) 2
regulation of snRNA transcription by RNA... 2
positive regulation of snRNA transcription... 2
nuclear polyadenylation-dependent mRNA... 2
polyadenylation-dependent mRNA catabolic... 2
U1 snRNA 3'-end processing (GO:0034473) 2
U5 snRNA 3'-end processing (GO:0034476) 2
ncRNA 3'-end processing (GO:0043628) 3
maturation of 5.8S rRNA (GO:0000460) 3
snRNA metabolic process (GO:0016073) 4
RNA splicing, via transesterification... 5
RNA splicing, via transesterification... 5
mRNA splicing, via spliceosome (GO:0000398) 5
rRNA metabolic process (GO:0016072) 5
rRNA processing (GO:0006364) 5
ribonucleoprotein complex biogenesis... 6
ncRNA processing (GO:0034470) 6
ncRNA metabolic process (GO:0034660) 7
mRNA processing (GO:0006397) 8
RNA splicing (GO:0008380) 8
mRNA metabolic process (GO:0016071) 10
positive regulation of macromolecule... 12
positive regulation of RNA biosynthetic... 12
positive regulation of transcription, DNA-... 12
positive regulation of nucleic acid-... 12
positive regulation of nucleobase-containing... 13
positive regulation of RNA metabolic... 13
RNA processing (GO:0006396) 13
RNA metabolic process (GO:0016070) 15
organic cyclic compound metabolic process... 16
cellular aromatic compound metabolic... 16
heterocycle metabolic process (GO:0046483) 16
nucleobase-containing compound metabolic... 16
nucleic acid metabolic process (GO:0090304) 16
gene expression (GO:0010467) 19
cellular nitrogen compound metabolic... 20
cellular metabolic process (GO:0044237) 26
primary metabolic process (GO:0044238) 26
nitrogen compound metabolic process... 26
organic substance metabolic process... 27
macromolecule metabolic process... 27
metabolic process (GO:0008152) 28
B
Lung Cancer
Counts
regulation of snRNA transcription by RNA... 2
positive regulation of snRNA transcription... 2
protein K11-linked ubiquitination... 3
protein polyubiquitination (GO:0000209) 5
RNA splicing, via transesterification... 7
RNA splicing, via transesterification... 7
mRNA splicing, via spliceosome (GO:0000398) 7
mRNA processing (GO:0006397) 10
RNA splicing (GO:0008380) 10
mRNA metabolic process (GO:0016071) 11
RNA processing (GO:0006396) 12
RNA metabolic process (GO:0016070) 15
gene expression (GO:0010467) 17
nucleic acid metabolic process (GO:0090304) 18
organic cyclic compound metabolic process... 19
cellular aromatic compound metabolic... 19
heterocycle metabolic process (GO:0046483) 19
nucleobase-containing compound metabolic... 19
cellular nitrogen compound metabolic... 23
macromolecule metabolic process... 26
organic substance metabolic process... 28
primary metabolic process (GO:0044238) 28
nitrogen compound metabolic process... 28
metabolic process (GO:0008152) 30
cellular metabolic process (GO:0044237) 30
C
Stomach Cancer
Counts
snRNA metabolic process (GO:0016073) 4
mRNA metabolic process (GO:0016071) 8
RNA metabolic process (GO:0016070) 12
D
Prostate Cancer
Counts
positive regulation of snRNA transcription... 2
mRNA processing (GO:0006397) 7
RNA splicing (GO:0008380) 7
mRNA metabolic process (GO:0016071) 8
RNA processing (GO:0006396) 10
negative regulation of molecular function... 11
RNA metabolic process (GO:0016070) 13
nucleic acid metabolic process (GO:0090304) 14
gene expression (GO:0010467) 16
organic cyclic compound metabolic process... 17
cellular aromatic compound metabolic... 17
heterocycle metabolic process (GO:0046483) 17
nucleobase-containing compound metabolic... 17
cellular nitrogen compound metabolic... 22
macromolecule metabolic process... 23
organic substance metabolic process... 27
cellular metabolic process (GO:0044237) 27
primary metabolic process (GO:0044238) 27
nitrogen compound metabolic process... 27

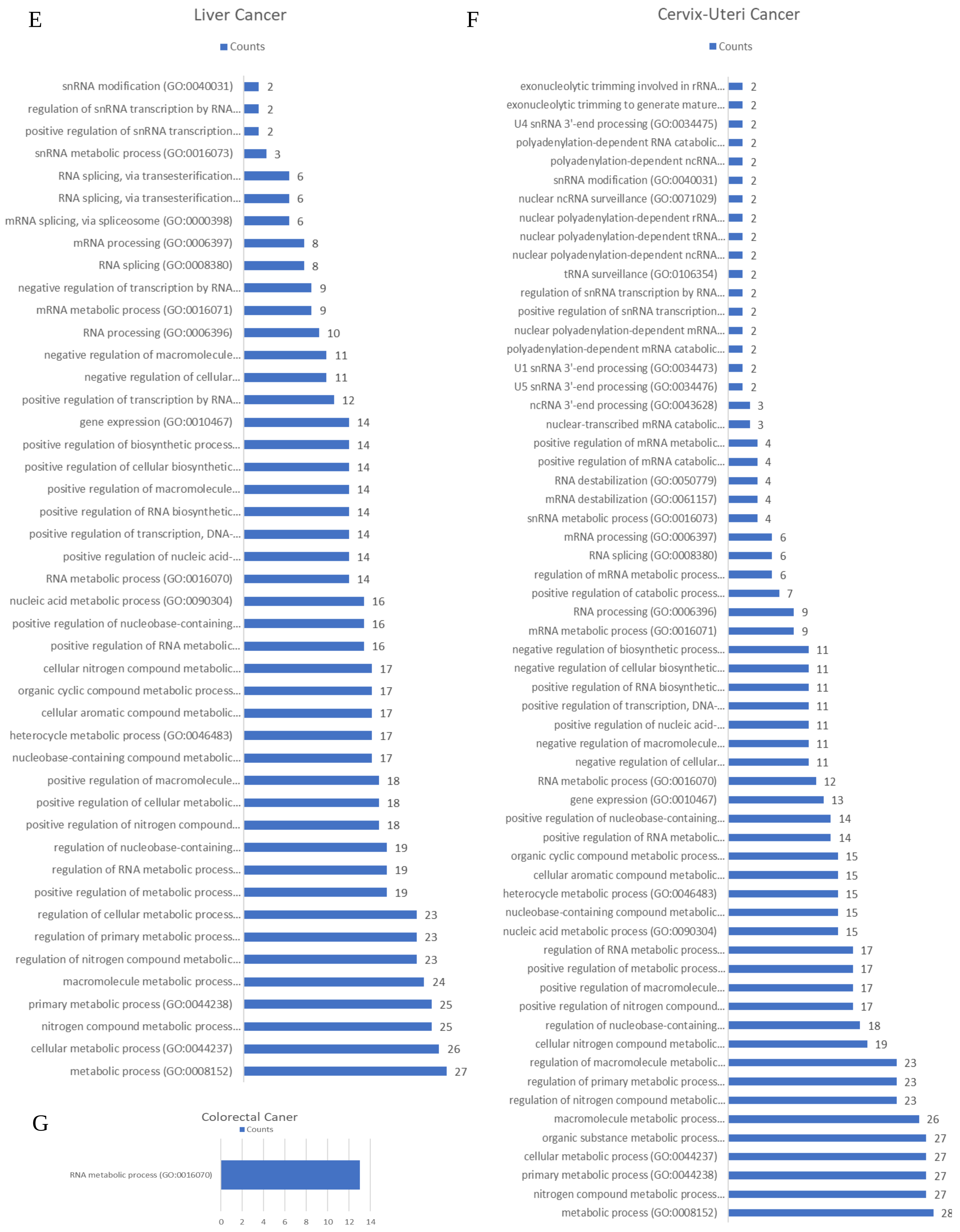

E
Liver Cancer
Counts
snRNA modification (GO:0040031) 2
regulation of snRNA transcription by RNA... 2
positive regulation of snRNA transcription... 2
snRNA metabolic process (GO:0016073) 3
RNA splicing, via transesterification... 6
RNA splicing, via transesterification... 6
mRNA splicing, via spliceosome (GO:0000398) 6
mRNA processing (GO:0006397) 8
RNA splicing (GO:0008380) 8
negative regulation of transcription by RNA... 9
mRNA metabolic process (GO:0016071) 9
RNA processing (GO:0006396) 10
negative regulation of macromolecule... 11
negative regulation of cellular... 11
positive regulation of transcription by RNA... 12
gene expression (GO:0010467) 14
positive regulation of biosynthetic process... 14
positive regulation of cellular biosynthetic... 14
positive regulation of macromolecule... 14
positive regulation of RNA biosynthetic... 14
positive regulation of transcription, DNA-... 14
positive regulation of nucleic acid-... 14
RNA metabolic process (GO:0016070) 14
nucleic acid metabolic process (GO:0090304) 16
positive regulation of nucleobase-containing... 16
positive regulation of RNA metabolic... 16
cellular nitrogen compound metabolic... 17
organic cyclic compound metabolic process... 17
cellular aromatic compound metabolic... 17
heterocycle metabolic process (GO:0046483) 17
nucleobase-containing compound metabolic... 17
positive regulation of macromolecule... 18
positive regulation of cellular metabolic... 18
positive regulation of nitrogen compound... 18
regulation of nucleobase-containing... 19
regulation of RNA metabolic process... 19
positive regulation of metabolic process... 19
regulation of cellular metabolic process... 23
regulation of primary metabolic process... 23
regulation of nitrogen compound metabolic... 23
macromolecule metabolic process... 24
primary metabolic process (GO:0044238) 25
nitrogen compound metabolic process... 25
cellular metabolic process (GO:0044237) 26
metabolic process (GO:0008152) 27
F
Cervix-Uteri Cancer
Counts
exonucleolytic trimming involved in rRNA... 2
exonucleolytic trimming to generate mature... 2
U4 snRNA 3'-end processing (GO:0034475) 2
polyadenylation-dependent RNA catabolic... 2
polyadenylation-dependent ncRNA... 2
snRNA modification (GO:0040031) 2
nuclear ncRNA surveillance (GO:0071029) 2
nuclear polyadenylation-dependent rRNA... 2
nuclear polyadenylation-dependent tRNA... 2
nuclear polyadenylation-dependent ncRNA... 2
tRNA surveillance (GO:0106354) 2
regulation of snRNA transcription by RNA... 2
positive regulation of snRNA transcription... 2
nuclear polyadenylation-dependent mRNA... 2
polyadenylation-dependent mRNA catabolic... 2
U1 snRNA 3'-end processing (GO:0034473) 2
U5 snRNA 3'-end processing (GO:0034476) 2
ncRNA 3'-end processing (GO:0043628) 3
nuclear-transcribed mRNA catabolic... 3
positive regulation of mRNA metabolic... 4
positive regulation of mRNA catabolic... 4
RNA destabilization (GO:0050779) 4
mRNA destabilization (GO:0061157) 4
snRNA metabolic process (GO:0016073) 4
mRNA processing (GO:0006397) 6
RNA splicing (GO:0008380) 6
regulation of mRNA metabolic process... 6
positive regulation of catabolic process... 7
RNA processing (GO:0006396) 9
mRNA metabolic process (GO:0016071) 9
negative regulation of biosynthetic process... 11
negative regulation of cellular biosynthetic... 11
positive regulation of RNA biosynthetic... 11
positive regulation of transcription, DNA-... 11
positive regulation of nucleic acid-... 11
negative regulation of macromolecule... 11
negative regulation of cellular... 11
RNA metabolic process (GO:0016070) 12
gene expression (GO:0010467) 13
positive regulation of nucleobase-containing... 14
positive regulation of RNA metabolic... 14
organic cyclic compound metabolic process... 15
cellular aromatic compound metabolic... 15
heterocycle metabolic process (GO:0046483) 15
nucleobase-containing compound metabolic... 15
nucleic acid metabolic process (GO:0090304) 15
regulation of RNA metabolic process... 17
positive regulation of metabolic process... 17
positive regulation of macromolecule... 17
positive regulation of nitrogen compound... 17
regulation of nucleobase-containing... 18
cellular nitrogen compound metabolic... 19
regulation of macromolecule metabolic... 23
regulation of primary metabolic process... 23
regulation of nitrogen compound metabolic... 23
macromolecule metabolic process... 26
organic substance metabolic process... 27
cellular metabolic process (GO:0044237) 27
primary metabolic process (GO:0044238) 27
nitrogen compound metabolic process... 27
metabolic process (GO:0008152) 28
G
Colorectal Caner
Counts
RNA metabolic process (GO:0016070)
0
2
4
6
8
10
12
14

Supplementary Figure S4. Gene ontology analysis for biological processes of model genes in different cancer types. (A) Breast cancer, (B) Lung cancer, (C)Stomach cancer (D)Prostate cancer (E)Liver cancer, (F) Cervix uteri cancer and (G) Colorectal cancer. The X-axis represents counts and Y-axis shows the GO term/Biological processes.
