# Supplementary figures and images for: Role of FRG1 in predicting the overall survivability in cancers using multivariate based optimal model
Source: Sci Rep. 2021 Nov 18;11:22505. doi: 10.1038/s41598-021-01665-w (PMC8602605; doi:10.1038/s41598-021-01665-w)

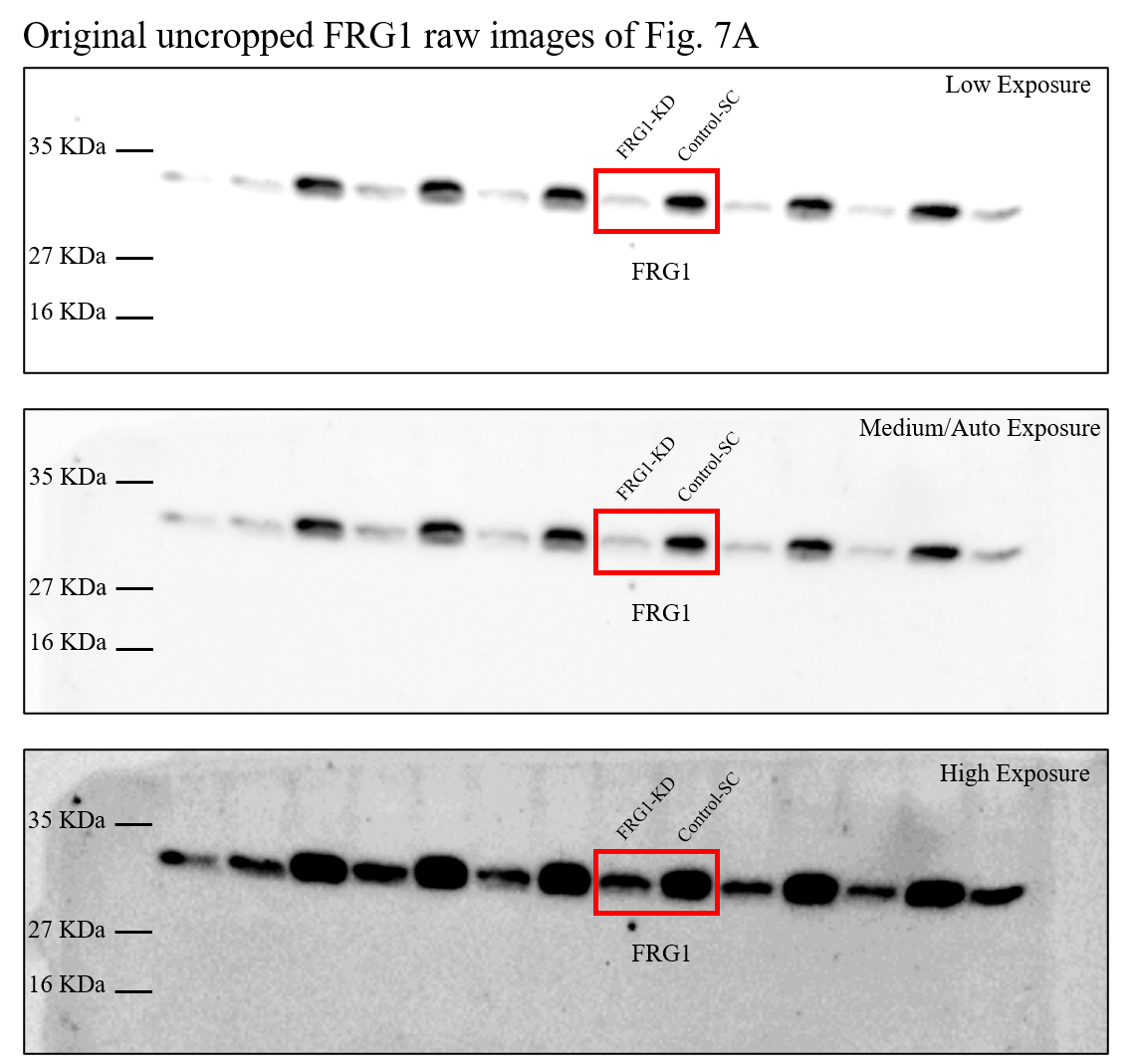

Supplement: Supplementary file 2 — Supplementary Information 2. [file 41598_2021_1665_MOESM2_ESM.tif]

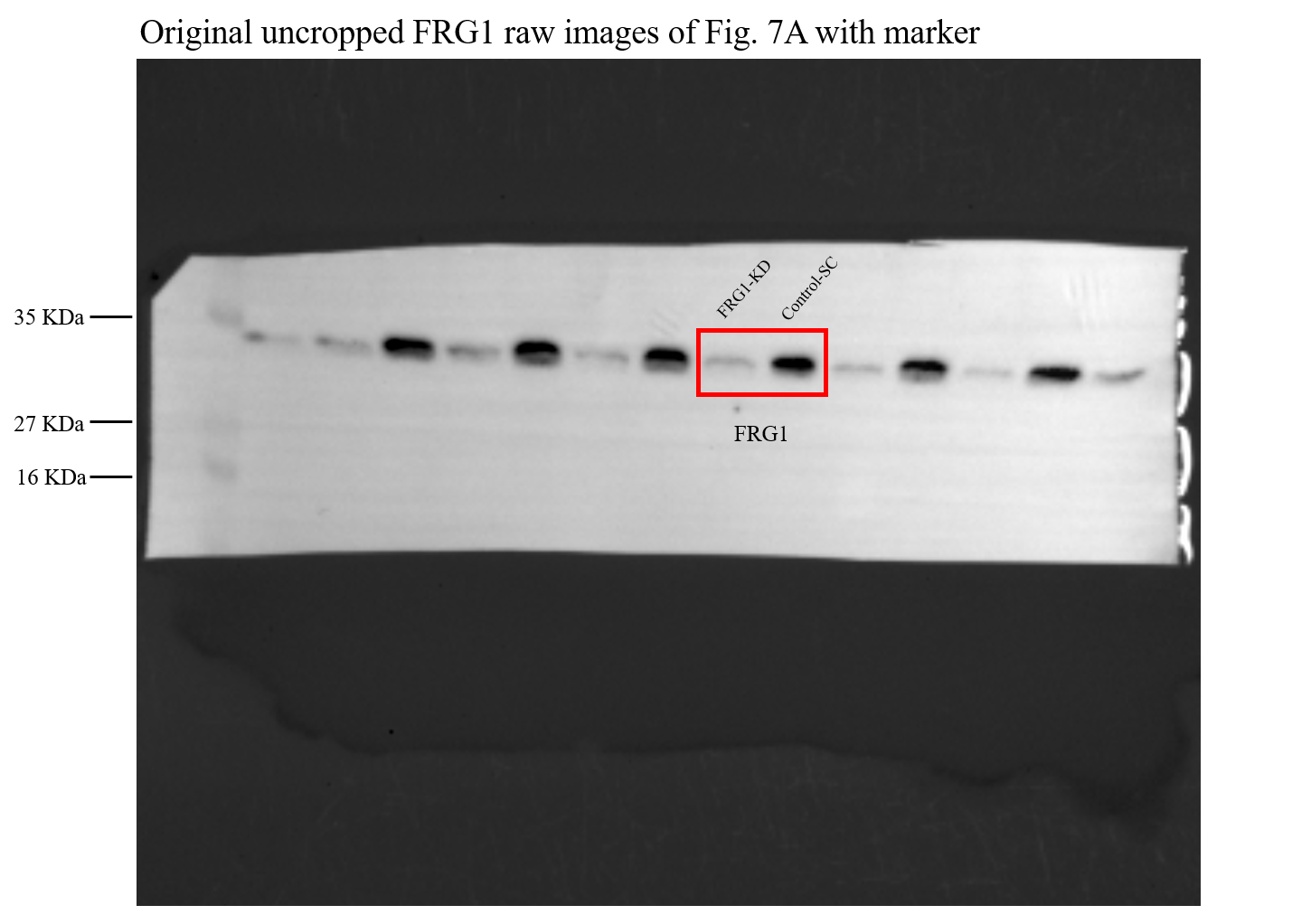

Supplement: Supplementary file 3 — Supplementary Information 3. [file 41598_2021_1665_MOESM3_ESM.tif]

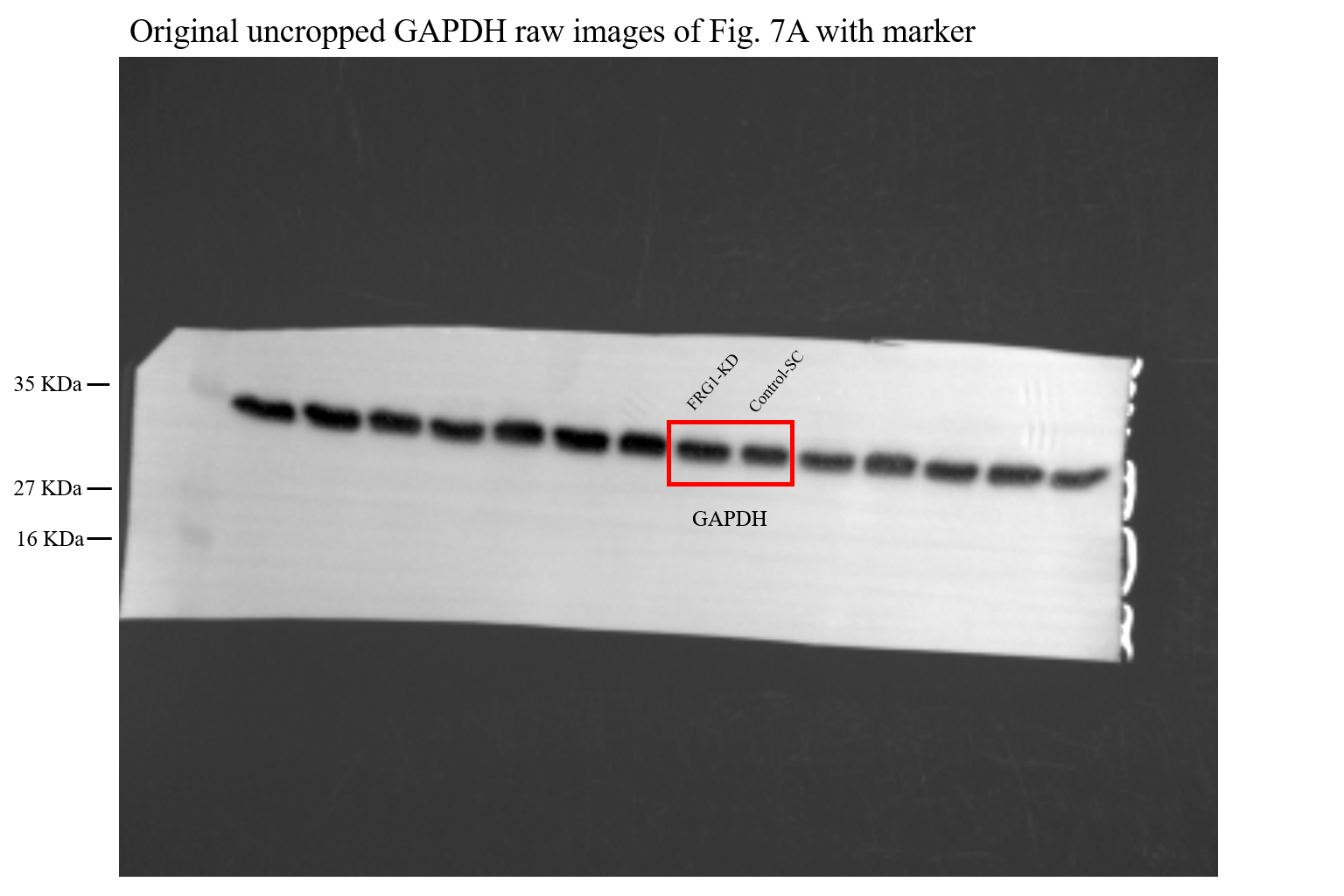

Supplement: Supplementary file 4 — Supplementary Information 4. [file 41598_2021_1665_MOESM4_ESM.tif]

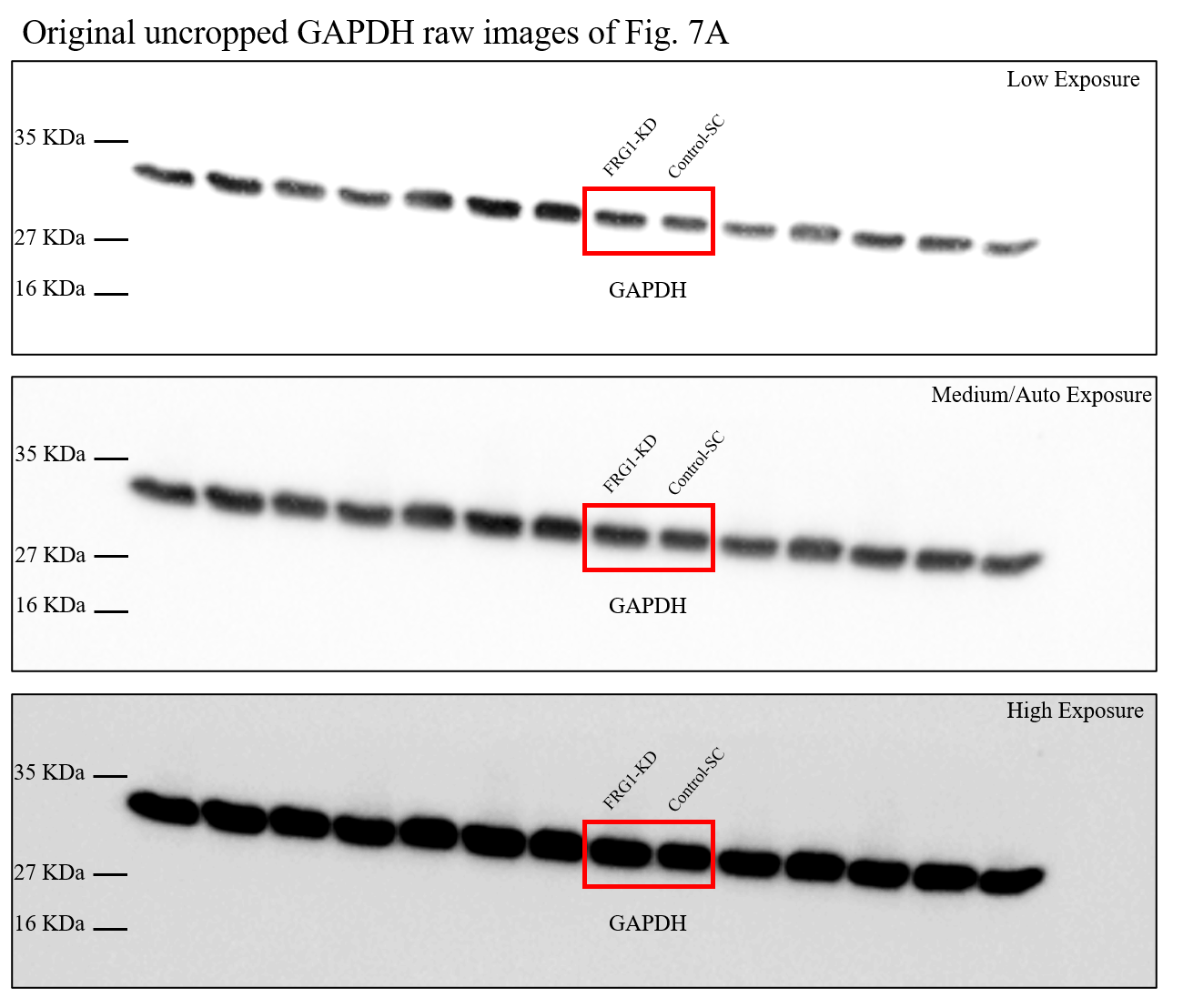

Supplement: Supplementary file 5 — Supplementary Information 5. [file 41598_2021_1665_MOESM5_ESM.tif]
